# Inborn errors of metabolism: Lessons from iPSC models

**DOI:** 10.1007/s11154-021-09671-z

**Published:** 2021-07-09

**Authors:** Rubén Escribá, Raquel Ferrer-Lorente, Ángel Raya

**Affiliations:** 1 Regenerative Medicine Program, Institut d’Investigació Biomèdica de Bellvitge ‐ IDIBELL, L’Hospitalet de Llobregat, Spain; 2 Program for Clinical Translation of Regenerative Medicine in Catalonia – P-[CMRC], L’Hospitalet de Llobregat, Spain; 3 Center for Networked Biomedical Research On Bioengineering, Biomaterials and Nanomedicine (CIBER-BBN), Barcelona, Spain; 4 Institució Catalana de Recerca I Estudis Avançats (ICREA), Barcelona, Spain

**Keywords:** Disease modeling, Human induced pluripotent stem cells, Reprogramming, Lysosomal storage disorders, CRISPR/Cas9, Targeted genome edition

## Abstract

The possibility of reprogramming human somatic cells to pluripotency has opened unprecedented opportunities for creating genuinely human experimental models of disease. Inborn errors of metabolism (IEMs) constitute a greatly heterogeneous class of diseases that appear, in principle, especially suited to be modeled by iPSC-based technology. Indeed, dozens of IEMs have already been modeled to some extent using patient-specific iPSCs. Here, we review the advantages and disadvantages of iPSC-based disease modeling in the context of IEMs, as well as particular challenges associated to this approach, together with solutions researchers have proposed to tackle them. We have structured this review around six lessons that we have learnt from those previous modeling efforts, and that we believe should be carefully considered by researchers wishing to embark in future iPSC-based models of IEMs.

**Supplementary Information:**

The online version contains supplementary material available at 10.1007/s11154-021-09671-z.

## Introduction

Inborn errors of metabolism (IEMs) are heritable monogenic disorders that often present congenitally with multi-system manifestations affecting various organs. Commonly, gene mutations in IEMs lead to inadequate enzyme or transporter activity and result in the accumulation of toxic substrates or a deficiency of essential metabolites. Although individually rare, IEMs are collectively quite common, with an estimated incidence of 1 in 4000 newborns [1, 2]. Even though various therapeutic approaches, including replacement therapy and small molecule drugs, have proven effective to treat several IEMs, there is still no cure for most of them, emphasizing the critical need for developing disease-modifying strategies that can ameliorate or halt disease progression.

Gaining a better understanding of the pathophysiological bases underlying IEMs is absolutely critical in order to develop new therapeutic agents. Animal models have provided invaluable insights into the pathophysiology of IEMs, but there remain questions of translatability to humans due to interspecies differences in physiology [3–6]. Additionally, recapitulating key phenotypes of IEMs *in vivo* has been challenging because transgenic animal models of these disorders are often nonviable or show early mortality [7]. Because of the limited availability of disease-relevant patients' samples (especially for rare monogenic diseases with limited number of patients available worldwide and limited access to the affected tissues), there is an urgent need for the development of human cellular models that recapitulate the most salient features of IEMs in order to investigate disease progression and to develop new therapeutic approaches. In this sense, the advent of induced pluripotent stem cell (iPSC) technology [8] has opened new perspectives for modeling human diseases. The possibility to reprogram human somatic cells to pluripotency offers an unprecedented opportunity to generate disease-specific iPSCs [9–12], for the development of cell-based experimental models.

In this review, we focus on the use of iPSC technology to model IEMs and how these models provide a valuable tool to set up innovative treatment approaches for particular IEMs. We discuss published iPSC models of inherited metabolic diseases and summarize the main lessons learnt from these efforts.

## Induced pluripotent stem cells as a powerful tool for disease modeling

In 2006, Takahashi and Yamanaka published a major technological breakthrough in the field of disease modeling, describing a method for reprogramming terminally differentiated adult dermal fibroblasts into cells with a gene expression profile and developmental potential similar to those of embryonic stem cells (ESCs) [8, 13, 14].

iPSC technology offers the possibility to generate patient-specific cells for modeling human diseases. While animal-based models have provided valuable insights into the pathogenic mechanisms of numerous human diseases [15–17], substantial species-specific differences prevent the recapitulation of key features characteristic of many human conditions [18–20]. Indeed, several rodent models have been described to not be adequate in the context of metabolic disorders: Lesch-Nyhan syndrome models do not recapitulate the common neurobehavioral aspects [21], vascular endothelial dysfunction is not recapitulated in Fabry disease models [22, 23], Niemman-Pick disease type C (NPC) models do not cover all aspects of the disease pathology [24], and the pathological phenotypes observed in sialidosis models appear to be shared across multiple lysosomal storage disorders (LSDs) and Alzheimer’s disease [25]. Similarly, the use of primary cultures of patient-derived cells, while helpful for studying disease etiology, is limited by the lack of expandable sources of patients’ cells, particularly for hard-to-access cells such as neurons and cardiomyocytes. Therefore, iPSC-based disease modeling appears as an attractive alternative to animal- and primary cell-based methods.

Identification of novel therapeutic approaches requires recapitulating key features of IEMs and relevant pathogenic mechanisms, a frequent limitation of conventional *in vitro* models. iPSC-based models can provide disease-related phenotypes in cell types relevant for the specific disease, thus representing a clear advantage over the use of fibroblasts or immortalized cell lines. For example, hiPSC-derived cells are more vulnerable to the toxic effects of substrate accumulation that are characteristic of several IEMs [26–28] and display a metabolic rate more similar to disease-relevant cells [29]. For inherited metabolic disorders affecting hepatocytes, immortalized cells lines are widely used. However, they do not resemble primary hepatocytes, have dysfunctional apoptotic pathways due to their carcinogenic nature and present restricted genotypic variability [30]. Similarly, primary hepatocytes, considered the gold standard for *in vitro* drug testing, gradually lose their hepatic functions during culture and are unable to proliferate [31]. Therefore, iPSC derivatives could provide an excellent alternative platform to overcome an important limitation associated with the identification of novel pharmacological treatments for IEMs.

Only fifteen years after the original description of induced reprogramming to pluripotency, enormous progress has been made in stem cell biology and regenerative medicine using iPSC technology [9, 24, 32–34]. Furthermore, iPSCs derived from healthy individuals and patients have accelerated advances in developing genuinely human experimental models of disease, novel pathogenic mechanisms or associated phenotypes have been elucidated [25, 26, 28, 35–37], new drugs initiated from iPSC-based screenings are in the pipeline [38–40] and the first clinical trials using human iPSC-derived products have been initiated [24, 41–43].

## Modeling inborn errors of metabolism *in vitro*

**Fig. 1:**
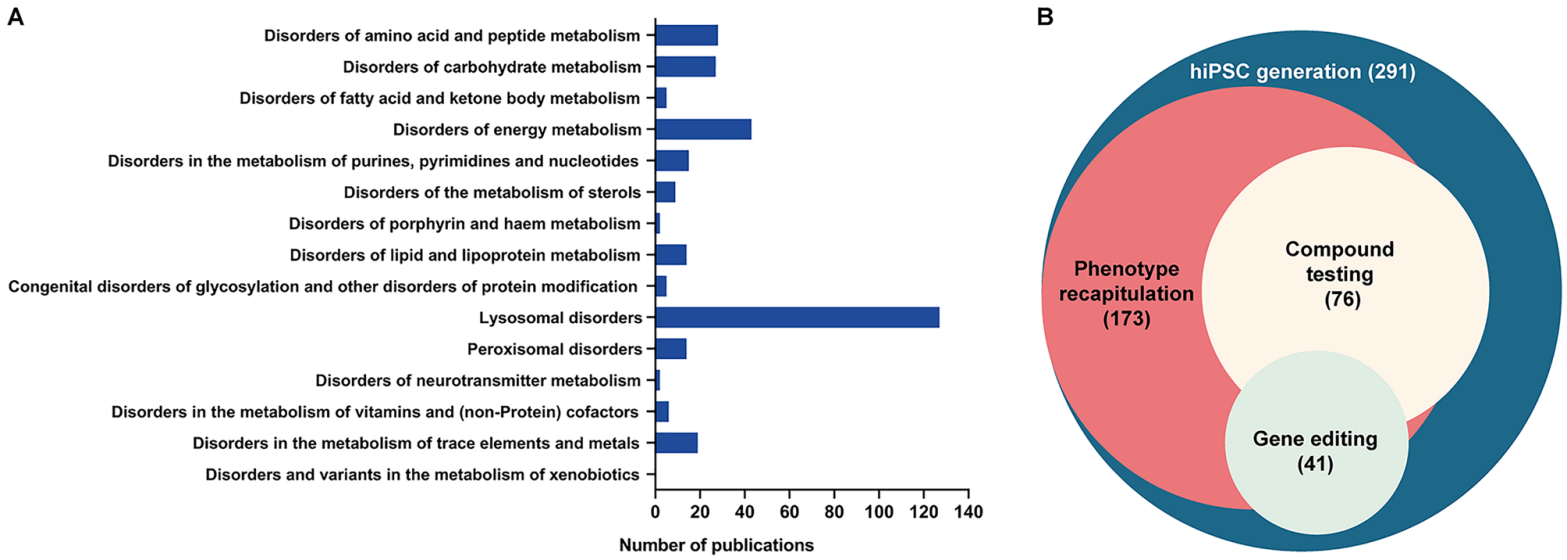
Analysis of published studies using iPSC technology for modeling of IEMs **A** Number of publications related to each of the fifteen main IEM groups as classified by the Society for the Study of Inborn Errors of Metabolism (SSIEM; classification available at www.ssiem.org/images/centralstore/resources/SSIEMClassificationIEM2011.pdf). **B** Distribution of the 291 publications reviewed for the current study, indicating those that reported recapitulation of phenotypic features of IEMs, screening of novel compounds, and the combination of iPSC technology with gene editing techniques

IEMs are a class of heterogeneous and rare diseases commonly caused by a defect in metabolic proteins, usually an enzyme or a transporter, that result in accumulation of harmful metabolites or deficiency of particular metabolites needed for cellular homeostasis [44]. Their classification can be difficult because of the multitude and variety of metabolic pathways involved. According to the hierarchical classification set forth by the Society for the Study of Inborn Errors of Metabolism (SSIEM), IEMs comprise 612 diseases with OMIM numbers, categorized into 15 main groups (www.ssiem.org/resources/resources/inborn-errors-classification). Although disease modeling remains a challenge in the context of IEMs, to date, dozens of these disorders have been modeled to some extent using patient-specific iPSC. We have identified from scientific literature a total of 107 IEMs for which iPSC lines have been generated, referenced with a total of 291 publications (Supplemental Table 1), among which lysosomal disorders have attracted special attention (Fig. 1A). In combination with specific protocols for differentiating patient-specific iPSC into disease-relevant cell types, these models are able to recapitulate key phenotypic features of IEMs and provide insight into disease mechanisms, as well as aiding in the identification of novel drug targets (Fig. 1B). Some of the most successful efforts at IEM modeling, together with the main lessons learnt from them, are discussed below.

### First lesson: iPSCs vs fibroblasts, highlighting the importance of the disease-relevant cell type

**Fig. 2:**
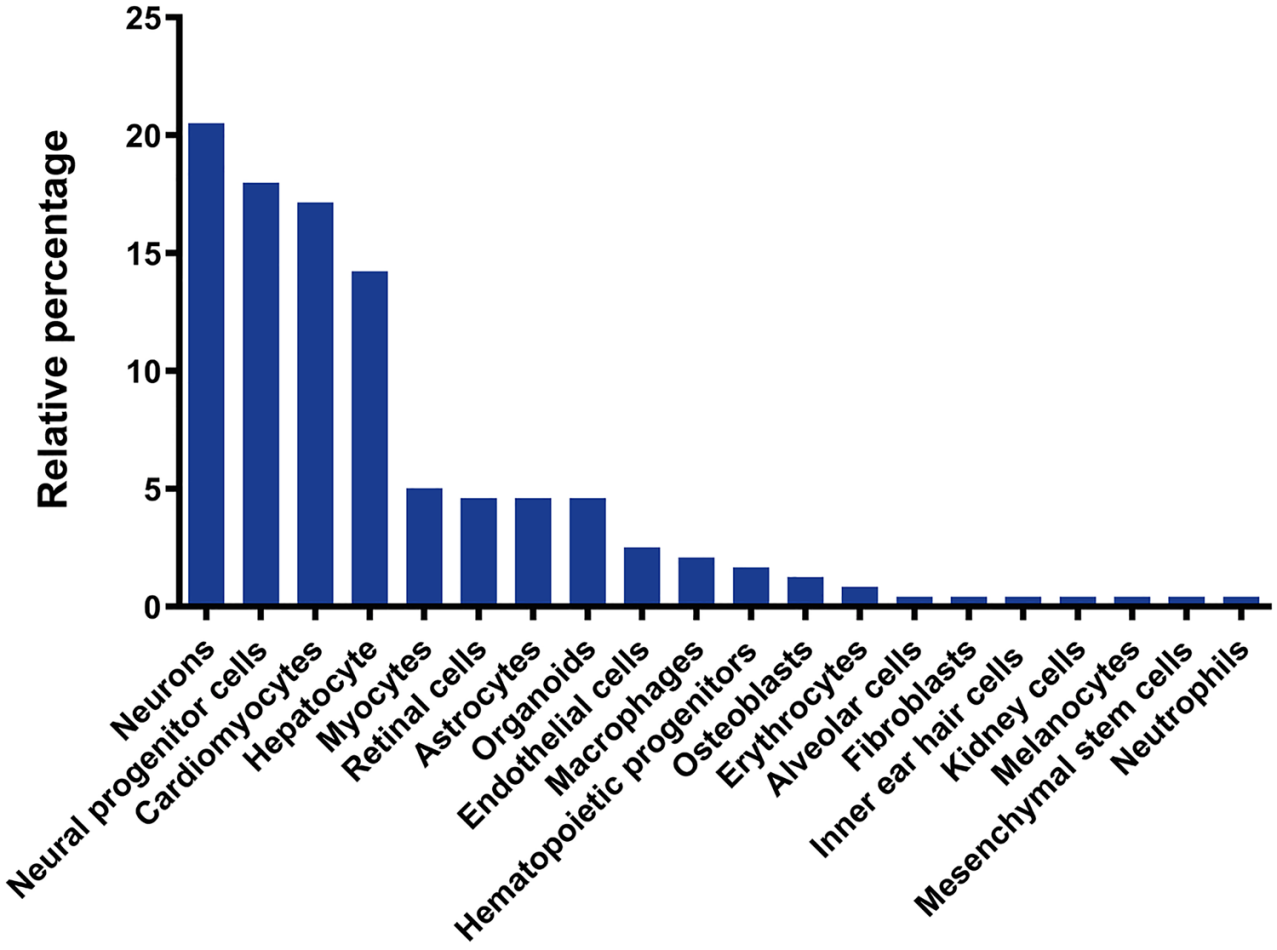
Main cell types differentiated from hiPSC for modeling of IEMs. Pie chart indicating the main cell types differentiated from hiPSC and used for disease modeling of IEMs in the reviewed literature, displayed following alphabetical order in clockwise direction. Out of the 20 different cell types identified in the relevant literature, neural derivatives and cardiomyocytes were used in over 50% of the published studies

For most IEMs, disease-relevant phenotypes cannot be investigated in fibroblasts, highlighting the importance of developing iPSC-derived models (Fig. 2). Nonetheless, in some cases, fibroblasts display disease-related phenotypes and thus, engaging into somatic cell reprogramming and subsequent iPSC differentiation might appear unnecessary. Several studies carried out with patients’ fibroblasts have contributed to our understanding of the pathogenic mechanisms of diseases [45], and provided valuable readouts for screening novel compounds [46–48]. Using this approach, Lee et al. reported on the key role of vascular endothelial growth factor (VEGF) and sphingosine kinase (SphK) in NPC pathogenesis [45]. The activity of SphK, a crucial enzyme in modulating the levels of sphingosine, was significantly decreased in fibroblasts from NPC patients, leading to abnormal sphingosine storage and autophagic defects. This dysfunction of SphK activity was linked to decreased VEGF expression. Thus, VEGF treatment ameliorated the NPC phenotype in patients’ fibroblasts and increased SphK activity. Several studies carried out with NPC fibroblasts, which showed an accumulation of unesterified cholesterol and lipids in lysosomal storage organelles, reported the evaluation of compound efficacy [46–48]. The benefit of compounds such as β-cyclodextrins, histone deacetylase inhibitors or rapamycin has been confirmed using NPC patient-derived fibroblasts. However, later studies found differences in drug responses between NPC neuronal cells and dermal fibroblasts even though they were derived from the same NPC patient. The application of human NPC neural stem cells as a cell-based disease model established that six out of the nine compounds reported as efficacious in NPC, fibroblasts, and mouse models, did not have significant effects in these cells [29]. Using the appropriate molecular and biochemical context is also fundamental for deciphering new cell type-specific pathogenic mechanisms: Seol et al. provided the first evidence that lysosomal sialidase gene (NEU1) deficiency alters, not only lysosomal dynamics, but also autophagic activity [25]; Odaka et al. revealed defective presynaptic exocytosis and excessive enhancement of AMPA-evoked Ca^2+^ influx in sialidosis [26]; and Liedtke et al. uncovered possible differences between NPC1 and NPC2 that showed indistinguishable biochemical phenotypes [28], among others.

IEMs comprise metabolic defects that can either affect almost all tissues, such as disorders of glycosylation, or specific cell types in which the mutated enzyme plays a critical role. In this sense, patient’s fibroblast can provide valuable information for diseases in which the associated biochemical defects are ubiquitously altered in nearly all cell types or the underlying phenotypes of the disease can be observed systemically.

### Second lesson: Important metabolic defects underlie IEMs -but iPSCs do not seem to care

For most IEMs modeled through iPSC-based technology to date, the metabolic defects that characterize these diseases do not seem to affect iPSC generation or maintenance, thereby enabling successful modeling of those diseases. Nevertheless, in some particular cases, iPSC generation from diseased somatic cells can be challenging, and reprogramming efficiency may be compromised by their severe pathophysiological defects. As examples, the generation of iPSC colonies for Gaucher disease, Pearson syndrome, Mucopolysaccharidosis type IIIB (MPSIIIB), Pompe disease, or NPC was found to be reduced when compared with that of healthy somatic cells [29, 49–53]. In a study by Huang et al. reprogramming fibroblasts from Pompe disease patients was not possible unless they conditionally rescued the expression of alpha-glucosidase (GAA) [51]. Nevertheless, later studies [54–57] successfully generated iPSC from infantile-onset and late-onset Pompe disease patients without the need of using rescue-based strategies. Similarly, iPSC generation from MPSIIIB patients by Lemonnier et al. required the exogenous complementation of the enzymatic defect [52]. In this case, reprogramming of MPSIIIB cells was efficient, but patient iPSC proliferated poorly and were inefficiently cloned unless the missing enzyme (α-N-acetyl-glucosaminidase) was exogenously supplied [52]. Lastly, iPSC generation was also hampered in NPC [29]. The authors initially found that only a few iPSC-like colonies appeared during the reprogramming process, disappearing after 5–7 passages. The difficulty in NPC-iPSC generation was due to cholesterol accumulation in fibroblasts. Thus, after the treatment of NPC fibroblasts with tocopherol, a compound that reduces cholesterol accumulation, NPC-iPSC stable in culture for over 20 passages could be successfully established [29].

IEMs encompass a plethora of highly diverse disorders. The presence and severity of the disease can also vary within individuals carrying the same pathogenic mutation. In any case, the known IEM mutations observed in patients are not developmentally lethal, and the early stages during the embryonic development are not severely compromised. In this regard, although the reprogramming efficiency might occasionally be reduced, the metabolic defects underlying IEMs do not seem to affect the generation and maintenance of iPSCs, which has been accomplished for dozens of these disorders. This finding reinforces iPSC technology as an important experimental tool to provide valuable insights into the pathophysiology of these diseases, further encouraging researchers to apply this powerful approach for IEM modeling.

### Third lesson: Variability needs to be accounted for when using iPSC to model IEMs

A defining characteristic of pluripotent stem cells is their potential to differentiate into cells of all three germ layers. However, a limiting factor regarding the use of iPSCs is, precisely, the variability in their differentiation potential. Not only the inter-individual differences among iPSC may have a strong impact in the differentiation capacity toward a specific cell type, but also between hiPSC clones from the same individual [58, 59]. There have been several attempts to characterize the sources of such variability. Prolonged culture and maintenance of pluripotent stem cells has been shown to promote genomic alterations, most frequently on chromosomes 12 and 17, likely reflecting the selective pressure processes taking place in culture [60]. Frequent amplifications of the WNT3/WNT9B cluster in the 17q21 chromosomal region have also been found to have important functional consequences affecting neural differentiation [61]. More recently, a comprehensive examination of the major sources of genetic and phenotypic variations in iPSC lines was undertaken by genome-wide characterization of 711 iPSC lines derived from 301 healthy individuals, established by the Human Induced Pluripotent Stem Cells Initiative (HipSci). The authors were able to identify consistent inter-individual effects that explained more than 46% of the phenotypic variation among iPSC lines [62].

Irrespective of the origin, phenotypic interline variability of iPSCs complicates assigning specific genotypes to disease-related cellular phenotypes. In this sense, the use of targeted nucleases as gene-editing tools is increasingly being used to counteract such variability, enabling the generation of isogenic controls that only differ in the presence/absence of the genetic variants under study. The versatility of targeting almost any locus in the genome to generate the desired targeted genetic modification becomes essential for the interpretation of the results and to strengthen the relationship between the genotype and phenotype.

The earliest attempts at generating sequence-specific changes in IEMs-derived iPSC were established with BAC- or AAV-based vectors targeting the ornithine-δ-aminotransferase (*OAT*) and *HPRT1* gene [63, 64]. Engineered nucleases, such as TALENs and zinc-finger nucleases (ZFN) have also been used to introduce specific mutations in iPSCs to model several diseases, such as ubiquinone deficiency, pyruvate kinase deficiency, NPC or LDLR deficiency [65–68]. More recently, CRISPR/Cas9 has emerged as the leading genome-editing technique in the context of iPSC-based disease modeling. The advent of CRISPR/Cas9 technology has opened unprecedented opportunities for this purpose due to its simplicity [69]. Successful efforts have been made in modeling inherited hypercholesterolemia with iPSC-corrected isogenic controls. Omer et al. and Okada et al. used CRISPR/Cas9 to correct a 3-base pair homozygous deletion and a homozygous point mutation in the *LDLR* gene, respectively, restoring low-density lipoprotein-cholesterol (LDL-C) uptake [70–72]. Recent studies generated corrected-isogenic controls of Fabry- iPSC lines carrying heterozygous mutations in the *GLA* gene. Differentiated cardiomyocytes and vascular endothelial cells from gene-corrected iPSC lines displayed restored levels of α-gal A protein [73, 74]. CRISPR/Cas9-based modeling of Sandhoff disease has also been achieved using isogenic controls in which one of the mutant alleles in the *HEXB* gene was corrected. In this way, cerebral organoids from mutant and gene-corrected iPSC were developed, demonstrating that accumulation of GM2 ganglioside was diminished and the neuronal differentiation was not impaired in isogenic controls when compared with the mutant *HEXB* line [75].

The use of genetic engineering and iPSC technology is a powerful combination especially for ultra-rare diseases where patients’ samples for iPSC generation are particularly limited. In order to overcome this limitation, specific mutations can be introduced in healthy control iPSC. Although the complex genomic background of real patients is not recapitulated when using control iPSC, these efforts provide valuable insights into the pathogenesis of specific gene mutations [76, 77].

The generation of iPSCs from healthy and diseased individuals allows capturing the complex genomic background from each individual. Genomic variability may impact in the differentiation capacity towards the cell of interest or even in the observed phenotypes. Thus, formally ascribing the appearance of disease-related phenotypes to the presence of particular gene mutations often requires the analysis of isogenic control cells that only differ in the specific mutation of interest. Although the studies combining iPSCs and gene-editing techniques for the modeling of IEMs are still only a small fraction (Fig. 1B), there is an increasing trend towards the use of such techniques in recent years, highlighting the importance of genome edition to address causality between gene mutations/variants and disease phenotypes.

### Fourth lesson: High-content/throughput screens may be feasible, but still a long way to go

For decades, the identification of novel compounds for treating human conditions has relied on high-throughput screening systems primarily based on cellular assays. Most cell-based screening assays have used either immortalized tumor-derived cell lines, in which the desired molecular target is heterologously expressed, or primary cells that better resemble the functions and phenotypes found *in vivo* [78]. Although more physiologically and preclinically relevant than immortalized cells, disadvantages of primary cell cultures such as their limited expansion and thus availability, have precluded their widespread adoption in high-throughput screening of small compounds. Major efforts are now aimed toward the use of iPSCs in drug discovery as they provide theoretically unlimited, homogeneous cell populations for cell type-specific compound screening, profiling and optimization [40, 79].

Although iPSC technology has facilitated the understanding of disease pathogenesis and the impact of compound modulators, drug development for monogenic diseases, including those related to IEMs, is still challenging [34, 80, 81]. In the field of lysosomal storage disorders much attention has been directed to target-based drug testing. Lysosomal accumulation of sphingolipids or cholesterol esters has been observed in iPSC-based models of Tay-Sachs, Niemann-Pick disease type A, B and C, Neural Ceroid Lipofuscinoses, and Wolman disease. In these studies, treatment with tocopherols and hydroxypropyl-cyclodextrins ameliorate lysosomal cholesterol and lipid accumulation, indicating their potential therapeutic use for these diseases [29, 67, 82–86]. Gaucher disease is arguably the most characterized iPSC model of lysosomal disorders, probably owing to the fact that mutations in the glucocerebrosidase gene (GBA1) constitute an important genetic risk factor for Parkinson’s disease (PD) [87]. In recent years, several target-based compound screens have been performed to identify molecules capable of decreasing the accumulation of glucosylsphingosine. In a study by Kim et al. inhibition of acid ceramidase by carmofur resulted, not only in the reduction of glucosylsphingosine levels, but also in a decrease in oxidized α-synuclein levels in GBA1-PD patient-derived dopaminergic neurons [88]. More recently, a small molecule inhibitor of mTOR (Torin 1) was able to upregulate lysosomal biogenesis and to enhance the autophagic clearance of Gaucher neurons [89].

However, the use of iPSC-based models of IEMs for medium- and high-throughput screening approaches is scarce to date. This most likely reflects the inherent challenges in generating disease affected cell types at high scale and purity [40]. In a study from Cayo et al. iPSC-derived hepatocytes from familial hypercholesterolemia patients were used to perform a large screening of existing drugs that could potentially increase exogenous LDL trafficking to endosomes and reduce exogenous LDL-C levels, including apoB. A total of 2,320 existing small molecules were tested for their ability to reduce apoB levels. The authors found that cardiac glycosides, such as digoxin, proscillaridin or ouabain, could lower the levels of apoB in culture medium by promoting its proteolytic turnover [90]. In a second high-content drug screening, the same laboratory generated a deoxyguanosine kinase (DGUOK) loss-of-function iPSC using CRISPR/Cas9 and differentiated them into hepatocyte-like cells [91]. DGUOK deficiency is the most common cause of mitochondrial DNA depletion syndrome, in which the production of deoxyadenosine monophosphate (dAMP) and deoxyguanosine monophosphate (dGMP) is diminished mainly in the liver. Using the same drug collection library, the authors interrogated the compounds for their ability to restore ATP levels in DGUOK-deficient hepatocytes. They identified NAD^+^ as a potential drug candidate that could increase not only ATP levels, but also the expression of all mitochondrial-encoded electron transport chain genes examined* in vitro* and *in vivo* [91]. Recently, iPSC-derived neurons have been used in two high-content drug screenings. In one study, neuronal progenitor cells from GM1 gangliosidosis were tested with 2.217 already approved drugs. Two compounds, amodiaquine and thiethylperazine were capable of activating autophagy both *in vitro* and *in vivo* [92]. In a second study, Lesch-Nyhan-derived neural progenitor cells were screened with 3.838 pharmacological compounds for their ability to increase cell viability. The authors identified 6 adenosine-like compounds that ameliorated cell survival and neurogenesis in HGPRT-deficient cells [93].

Over the past few years, compound-screening assays using iPSC-derived cells has emerged as a new powerful strategy for the pharmaceutical industry, facilitating target and lead identification, and lead optimization. However, there is still a long way to go in the context of IEMs. iPSCs are particularly important given that they can be reproducibly cultured in high abundance and give rise to different cell types from the same individual, which facilitates optimization for compound safety, selectivity and efficacy. In addition, the importance of stringent quality controls and well defined and established workflows at every stage of the process will be indispensable for the identification of novel compounds, and thus for the successful use of iPSC-based platforms for drug discovery and preclinical studies.

### Fifth lesson: iPSC-based technology opens the door to modeling pre-clinical stages of disease

Theoretically at least, almost any genetic disease lends itself to be modeled employing iPSC-based technology. However, this strategy has proved so far to be more amenable for tackling monogenic diseases associated to highly penetrant disease-causing mutations, as opposed to environmentally modulated, sporadic or diseases of unknown cause [94]. Congenital or early-onset diseases also appear to be more readily modeled *in vitro* using iPSC-based strategies than late-onset diseases. This could be linked to the fact that current iPSC differentiation protocols yield cells with immature or fetal-like characteristics, but it may reflect the difficulty of recapitulating aging, a major risk factor in many late-onset diseases, in standard laboratory conditions. These limitations notwithstanding, several studies have decribed the appearance of disease-related phenotypes in iPSC-based models of PD, Huntington disease, and Alzheimer’s disease [95, 96]. Although these disorders may appear late in life, aberrant molecular and cellular phenotypes are detectable in culture, illustrating the possibility to study early stages of disease progression.

Canals et al. generated an iPSC-based experimental model of Sanfilippo type C syndrome, a lysosomal storage disorder with progressive neurodegeneration [97]. Differentiated mature neurons were able to recapitulate the main known phenotypes of the disease, such as the accumulation of glycosaminoglycan (GAG) over time and abnormal vacuoles morphology. The authors found that neuronal GAG accumulation was not statistically significant until cells had been in culture for 9 weeks, in concordance with the progressive nature of the disease. However, the authors found that neuronal network activity and connectivity were already altered at 3 weeks of differentiation, suggesting that similar approaches could be used to detect early functional signs that predate the clinical onset of other diseases as well. If realized, this would facilitate the identification of potential therapeutic strategies that would prevent, rather than revert, the appearance of clinical signs of the disease in patients.

### Sixth lesson: Advanced culture systems, new tools to overcome the limitations of 2D cultures

One of the main challenges associated with iPSC-based experimental approaches is the relative immaturity of iPSC derivatives obtained with current differentiation protocols, which are typically carried out under standard two dimensional (2D) culture conditions [98, 99]. It is commonly thought that 2D cell culture systems lack critical information cues for cell differentiation/maturation, including tissue-specific architecture and biomechanical and biochemical signals originating from cell–cell and cell–matrix interactions [100]. Many of those information cues are present in organoid-type 3D culture systems, which offer levels of cell differentiation, tissue organization and response to drugs more closely resembling the *in vivo* situation [101, 102].

For example, Akbari et al. reported on the generation of functional hepatic organoids for Citrullinemia type I that recapitulated important disease-related phenotypes such as the decrease in ammonia detoxification capacity [103]. Similarly, a robust spinal cord organoid system derived from iPSCs of a MELAS (Mitochondrial encephalomyopathy, lactic acidosis and stroke episodes) patient was developed to further investigate the neurogenesis defects in this disease. Motor neurons, which could not be reproducibly obtained using conventional 2D differentiation protocols, were reproducibly generated in the organoid model [104]. More recently, a brain organoid model of Leigh syndrome, the most severe pediatric manifestation of mitochondrial diseases, revealed compromised neural morphogenesis due to defective metabolic state. This model offers hope for solving a major obstacle for developing effective therapies for this syndrome: the lack of suitable model systems recapitulating the human disease course. Thanks to this model, Inak et al. provided novel mechanistic insights and suggested potential interventional strategies for this incurable disease [105].

Organoids are valuable tools for precision medicine and, in combination with genetic-engineering approaches, can be used to directly test the role of pathogenic mutations in a controlled environment. Despite the numerous advantages of organoids over conventional 2D cell culture systems, some limitations remain, such as the relative lack of standardized protocols to generate organoids and the absence of vascularization and of complex inter-organ communication. To address current technical challenges in organoid research, the integration of organoids with microfluidic on-a-chip devices is emerging as an advanced, yet complementary, bioengineering approach. Organ-on-a-chip devices are able to control the biochemical and biophysical microenvironment, resulting in increased tissue maturation and vascularization, and offering a more *in vivo*-like phenotype with multi-organ capabilities for IEM disease modelling [106–108]. A pioneering adoption of on-a-chip approaches in the field of IEM modeling investigated the pediatric cardiomyopathy Barth syndrome. Combining gene editing and heart-on-a-chip technology, the authors were able to provide new insights into the disease mechanisms [108]. However, organ-on-a-chip devices are typically of very low throughput, limiting its applicability in the early stages of drug discovery [109].

## Concluding remarks

**Fig. 3:**
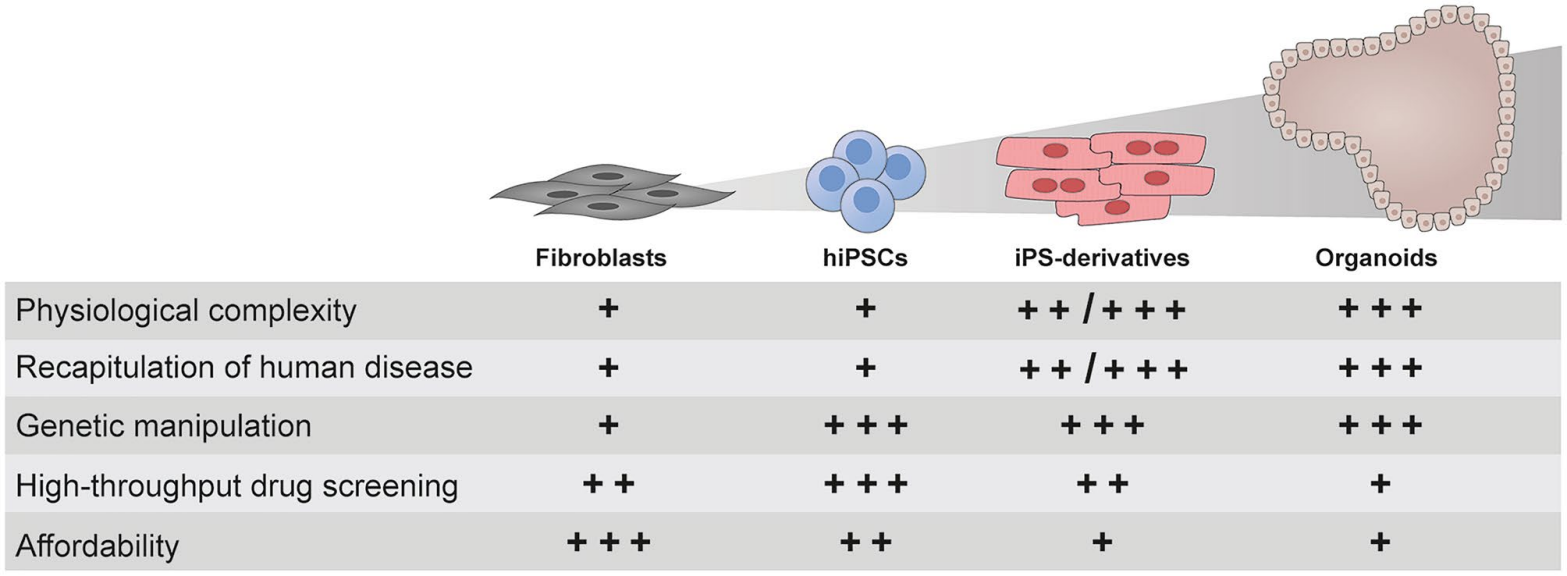
Comparison of different cell culture systems for modeling of IEMs. Patient-specific fibroblasts, along with their reprogrammed hiPSC and hiPSC-derivatives (in form of 2D cell cultures or organoid systems), are compared for their relative limitations and benefits. Score marks are represented as follows: ‘ + ’: not suitable; ‘ + + ’: good and ‘ + + + ’: optimal

**Fig. 4:**
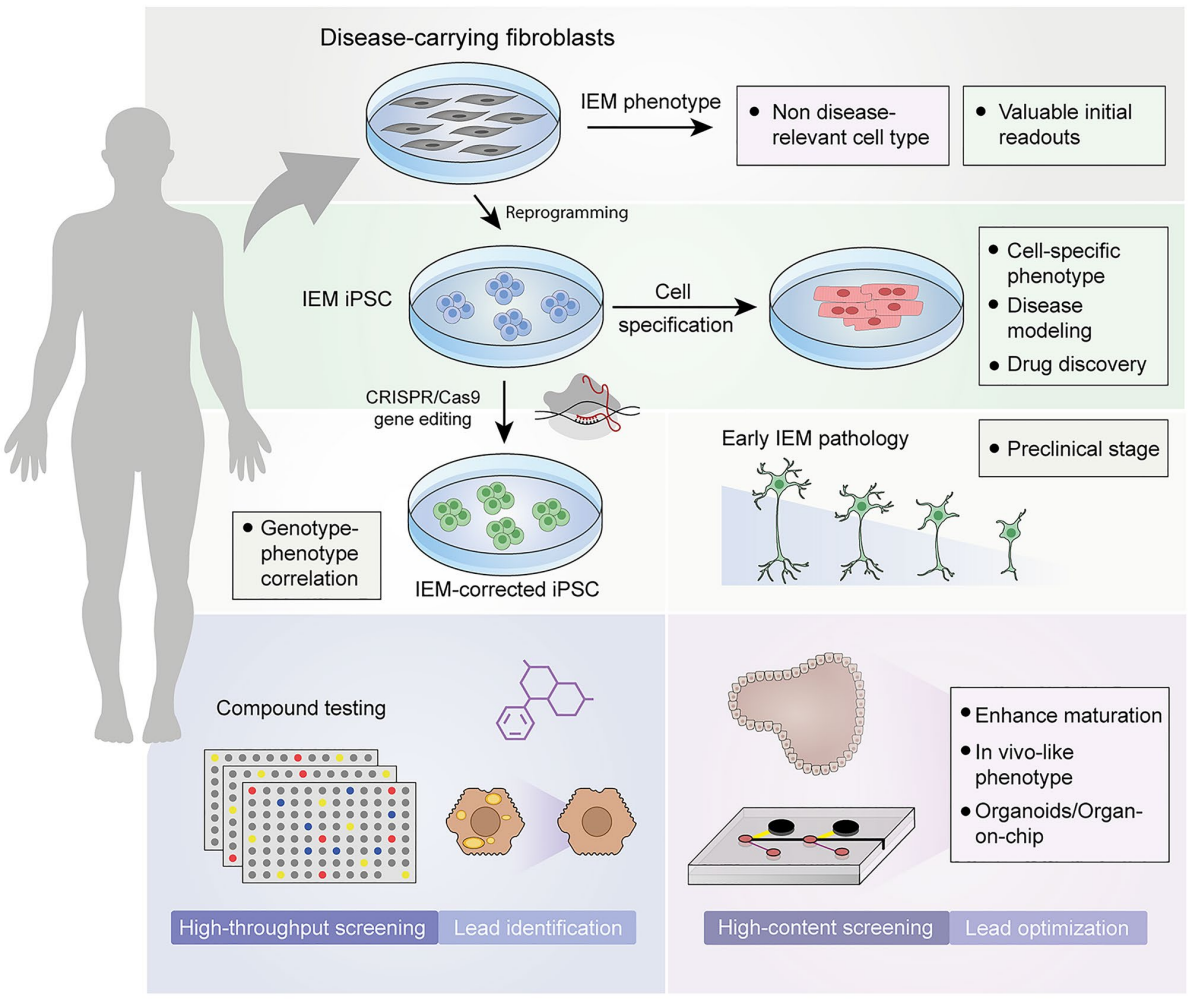
Schematic representation of the lessons learnt from iPSC models of IEMs. Somatic cells such as fibroblasts can be obtained from patients with IEMs to study pathogenic mechanisms, but in a cellular and metabolic context that may differ from that of disease-affected cells. Somatic cells can be reprogrammed into iPSC carrying the IEM-specific genetic alteration. iPSC can be differentiated into the disease-relevant cell type (such as cardiomyocytes to study disorders of energy metabolism or glycogen storage disorders). CRISPR/Cas9-based gene-editing technology allows the generation of isogenic sets of iPSC that can be used to properly associate genetic alterations to specific phenotypes. The combination of gene editing and iPSC technology is a remarkable tool to study preclinical stages (such as the neural defects in Sanfilippo C) and to perform high-throughput drug screenings (for example to reduce the hypercholesterolemia in iPSC-derived hepatocytes). The immature phenotype of iPSC derivatives is a current limitation in the field, which can be overcome by using advanced culture systems such as organoids and organ-on-a-chip devices, which generate cells more similar to their *in vivo* counterpart, making them suitable for high-content screenings and lead optimization

The ability to reprogram somatic cells to iPSCs is gradually changing the way we develop experimental disease models, especially for rare monogenic diseases, offsetting the limited number of patients available worldwide, the difficulty in gaining access to the mostly affected tissues, and the lack of bona fide experimental animal models. The combination of human iPSC-based technology and site-directed genome edition constitutes an exciting and powerful platform for complex disease modeling and drug screening. However, despite the promising advantages offered by combining these two technologies, important limitations and challenges need to be taken into account if disease models are to be exploited to their fullest (Fig. 3). Efforts at modeling human diseases in general, and IEMs in particular, have taught us already some lessons that we believe ought to be considered when devising future experimental models (Fig. 4): 1) iPSC technology emphasizes the importance of the disease-relevant cell types. Fibroblasts can provide valuable readouts for primary screening of novel compounds, but the differential response between fibroblasts and the specific cell type(s) affected in the disease highlights that, in many cases, iPSC-based cell models could be the preferred option for this purpose; 2) the characteristic metabolic defects that underlie IEMs do not seem to affect iPSC generation and maintenance, reinforcing iPSC technology as an important experimental tool to provide insights into the pathophysiology of IEMs; 3) the inter-line and inter-clone variability in differentiation potential of iPSCs needs to be accounted for when modeling IEM. A straightforward manner to address this limitation consists on the parallel interrogation of isogenic controls, which are currently easily generated thanks to CRISPR/Cas9-based genomic edition technology; 4) although still a long way to go in the context of IEMs, iPSCs-based models emerge as serious candidates to be used as medium- and high-throughput screening platforms since they can provide theoretically unlimited amounts of homogeneous, patient-specific, and disease-relevant cell types; 5) iPSC-based technology offers the possibility to model pre-clinical stages of late-onset diseases and to identify early functional signs that predate the clinical phenotypes. This pre-clinical time window could facilitate the development of potential therapeutic strategies that would prevent the appearance of clinical disease; and 6) Advanced culture systems hold remarkable potential for increasing the maturation state of iPSC derivatives, thus extending the validity of such disease models to phenotypes not easily attainable in conventional 2D culture systems.

In conclusion, carefully designed and properly controlled iPSC-based models of IEMs recapitulate key features of the disease phenotype and respond to pharmacologic manipulation predictably. As such, iPSC-based models of IEMs have enabled uncovering the role of novel pathogenic mechanisms in sialidosis and NPC, identifying new drug compounds potentially effective for DGUOK deficiency and hypercholesterolemia, and developing novel drug candidates and target pathways for GM1 gangliosidosis, among many other examples of success. Whereas remarkable progress has been made over the past decade, iPSC technology is still in its infancy and important challenges will need to be addressed. Of note, the functional immaturity of iPSC-derived cells is an important consideration when studying IEM and age-related diseases. As developments in iPSC technology itself progress towards better reproducibility and scalability, we believe that iPSC-based approaches will become a powerful tool for precision medicine, capable of predicting disease trajectories and potential effective treatments at the individual level, not only for IEMs but also applicable to many other diseases.

## Supplementary Information

Below is the link to the electronic supplementary material.

Supplementary file1 (PDF 424 KB)

## Abbreviations

2D: Two-dimensional
3D: Three-dimensional
CRISPR: Clustered regularly interspaced short palindromic repeats
dAMP: Deoxyadenosine monophosphate
dGMP: Deoxyguanosine monophosphate
DGUOK: Deoxyguanosine kinase
ESCs: Embryonic stem cells
FD: Fabry disease
GAA: Alpha-glucosidase
GAG: Glycosaminoglycan
GBA: Glucocerebrosidase
HipSci: Human Induced Pluripotent Stem Cells Initiative
IEMs: Inborn errors of metabolism
iPSCs: Induced pluripotent stem cells
LDL-C: Low-density lipoprotein-cholesterol
LSDs: Lysosomal storage disorders
MELAS: Mitochondrial encephalomyopathy, lactic acidosis and stroke episodes
MPSIIIB: Mucopolysaccharidosis type IIIB
NPC: Niemman-Pick disease type C
OAT: Ornithine-δ-aminotransferase
OMIM: Online mendelian inheritance in man
PD: Parkinson’s disease
SphK: Sphingosine kinase
SSIEM: Society for the Study of Inborn Errors of Metabolism
TALENs: Transcription activator-like effector nuclease
VEGF: Vascular endothelial growth factor
ZFN: Zinc-finger nucleases
