# Supplementary material for: Inborn errors of metabolism: Lessons from iPSC models
Source: Rev Endocr Metab Disord. 2021 Jul 9;22(4):1189–200. doi: 10.1007/s11154-021-09671-z (PMC8724155; doi:10.1007/s11154-021-09671-z)
Supplement: Supplementary file 1 — Supplementary file1 (PDF 424 KB) [file 11154_2021_9671_MOESM1_ESM.pdf]

Supplementary Information

Inborn errors of metabolism: Lessons from iPSC models

Rubén Escribá[1,2,3,#], Raquel Ferrer-Lorente[1,2,3,#], Ángel Raya[1,2,3,4,*]

[1]*Regenerative Medicine Program, Institut d'Investigació Biomèdica de Bellvitge - IDIBELL, L'Hospitalet de Llobregat, Spain.*

[2]*Program for Clinical Translation of Regenerative Medicine in Catalonia – P-[CMRC], L'Hospitalet de Llobregat, Spain*

[3]*Center for Networked Biomedical Research on Bioengineering, Biomaterials and Nanomedicine (CIBER-BBN), Barcelona, Spain.*

[4]*Institució Catalana de Recerca i Estudis Avançats (ICREA), Barcelona, Spain.*

# These authors contributed equally to this work.

* Correspondence should be addressed to: A.R.: araya@idibell.cat

Key words: disease modeling; human induced pluripotent stem cells; reprogramming; lysosomal storage disorders; CRISPR/Cas9; targeted genome edition;

Table Supplementary 1. Summary of induced pluripotent stem cell models for inborn errors of metabolism

Disease	OMIM number	Gene (Mutation)	Differentiated cell type	Disease phenotype described	Reference
**1. Disorders of amino acid and peptide metabolism**					
**1.1. Urea cycle disorders and inherited hyperammonaemias**					
1.1.1. Ornithine transcarbamylase deficiency	311250	**OTC** c.663+2T>G c.386G>A, r.299_386del and r.386_387ins386+1_384+4	Hepatic organoids	Deregulated urea cycle activity. Ureagenesis is decreased.	[1, 2]
1.1.2. Citrullinaemia type1	215700	**ASS1** Exon 6, c.364-2 A>G, p. G259*; Exon 13, c.910 C>T, p.R304W; c.1168G>A	Hepatocyte, hepatic organoids	Ureagenesis is decreased. Tricarboxylic acid (TCA) cycle metabolites are accumulated. Response to arginine treatment	[3, 4]
1.1.3. Argininosuccinic aciduria	207900	**ASL** c.557 G>A, p.R186Q; c.857 A>G, p. Q286R; c.655+1 G>A; c.857 A>G, p.Q286R	Endothelial cells	Decreased nitric oxide (NO) signalling. Increased oxidative stress. Impaired angiogenesis *in vitro* and *in vivo*	[5]
1.1.4. Citrullinemia Type 2	603859	**SLC25A13** 851del4, p.R284fs (286X); IVS1ins3kb, p.A84fsc (585X)	Hepatocyte	Lack of urea production. Increased triglyceride levels. Peroxisome proliferator-activated receptors-α downregulated. Aberrant mitochondrial β-oxidation and abnormal mitochondrial structure	[6]

**1.2. Organic acidurias**					
1.2.1. Propionic aciduria	232000	**PCCA** c.1899+4_1899+7delAGTA; p.C616_V633del; p.G477Efs*9	Cardiomyocytes	Reduced oxygen consumption. Accumulation of lipid droplets. Endoplasmic reticulum stress. Calcium perturbations	[7, 8]
		**PCCB** c.1218_1231del14ins12 (p.G407 fs)	Not performed	Not performed	[9, 10]
1.2.2. Methylglutaconic aciduria					
1.2.2.1. Methylglutaconic aciduria type II, Barth syndrome	302060	**TAZ** c.517delG; c.328 T>C, c.590 G>T; p.G197V, c.110-1; c.AG>AC; p.r.spl Ex2del?; c.170 G>T; p.R57L	Cardiomyocytes	Abnormal cardiolipids biogenesis and mitochondrial function. Metabolic alterations and energy production. Rearrangements of respiratory chain complexes. Increased ROS production. Abnormal sarcomerogenesis. Severe defect in contractility	[11-16]
1.2.2.2. Methylglutaconic aciduria type V	610198	**DNAJC19** IVS3-1G>C	Cardiomyocytes	Abnormal mitochondrial morphology. Mitochondrial dynamics imbalance	[17]
1.2.3. Aminoacylase deficiency					
1.2.3.1. Aminoacylase 2 deficiency (Canavan disease)	271900	**ASPA**	Neural precursor cells (NPCs)	Phenotype rescued after corrected NPCs transplantation into mouse model	[18]

**1.3. Disorders of the metabolism of branched-chain amino acids not classified as organic acidurias**					
1.3.1. Maple syrup urine disease					
1.3.1.1. BCKD E1 alpha subunit of deficiency	248600	**BCKDHA** c.1280_1282 delTGG and c.632C>T)	Not performed	Not performed	[19]
1.3.1.2. BCKD E1 beta subunit of deficiency	248600	**BCKDHB** c.502C>T/p.R168C, c.965C>T/p.T322I	Not performed	Not performed	[20]
**1.4. Disorders of phenylalanine or tyrosine metabolism**					
1.4.1. Phenylalanine hydroxylase deficiency	261600	**PAH** c.331 C>T; c.975 C>G	Not performed	Not performed	[21]
**1.5. Disorders of ornithine or proline metabolism**					
1.5.1. Ornithine aminotransferase deficiency	258870	**OAT** c.677 C>T; p.A226V	Retinal pigment epithelium	Very low OAT activity	[22, 23]
**1.6. Disorders of serine, glycine or glycerate metabolism**					
1.6.1. P protein deficiency	238300	**GLDC** c.1742C > G (p.Pro581Arg) and c.2368C > T (p.Arg790Trp)	Not performed	Not performed	[24]
**1.7. Disorders of amino acid transport**					
1.7.1. Lowe syndrome	309000	**OCRL** c.2582-1 G>T, c.2470-2 A>G; 2179delC, c.2626dupA	Neurons, kidney cells, neural progenitor cells	F-actin and WAVE-1 expression altered. Cytoskeletal disorganization	[25-28]

2. Disorders of carbohydrate metabolism

2.1. Disorders of glyoxylate metabolism

2.1.1.	Primary hyperoxaluria type I	260000	**AGXT**<br>c.731 T>C; p.I244T<br>c.508G>A (G170R) and c.364C>T (R122*)	Hepatocytes	Not performed	[29-32]

2.2. Glycogen storage disorders

2.2.1.	Glycogen storage disease type 1a, von Gierke	232200	**G6PC**<br>c.648G > T; p.Leu216	Hepatocyte	Intracellular glycogen accumulation, lipid accumulation and excessive production of lactic acid	[33, 34]
2.2.2.	Glycogen storage disease type 1b, von Gierke	232220	**G6PT**	Hepatocytes and neutrophils	Glycogen, lactate, pyruvate and lipid accumulation. Superoxide anion production. Increased annexin V binding. Activation of caspases 3 and 9	[35]
2.2.3.	Glycogen storage disease type II, Pompe	232300	**GAA**<br>del ex18/del ex18;<br>c.1935 C>A;<br>c.1935 C>A/c.2040 +1 G>T;<br>p.D645E/c.1935 C>A;<br>del Ex18/del525T;<br>c.1062 C>G;<br>p.Y354X/c.1935 C>A;<br>IVS1-13 T>G/ del Ex18;<br>1441delT/2237 G>A;<br>c.796 C>T/c.1316 T>A;<br>IVS1-13 T>G/ del525T;<br>IVS1-13 T>G/ c.923 A>T c.2560C > T (p.R854X); c.1822C>T, p.R608X; c.2662G>T, p.E888X	Cardiomyocytes, skeletal muscle cells, neural progenitors cells, neurons, hepatocytes	Increased lysosomal glycogen accumulation, mitochondrial dysfunction, multiple ultrastructure aberrances, large glycogen-containing vacuoles, glycan processing abnormality, increased oxidative stress, suppressed mTORC1 activation	[36-51]

2.2.4. Glycogen storage disease type V, McArdle	232600	**PYGM** c.148 C>T; p.R50Ter	Not performed	Not performed	[52]
2.2.5. Glycogen storage disease type IX					
2.2.5.1. Cardiac muscle phosphorylase kinase deficiency	261740	**PRKAG2** c.905 G>A; p.R302Q; p.R531Q; p.N488I	Cardiomyocytes	Cellular enlargement. Electrophysiological irregularities. Glycogen accumulation. AMPK activity increased. Arrhythmic calcium handling. Increased twitch force	[53-55]
**3. Disorders of fatty acid and ketone body metabolism**					
**3.1. Disorders of carnitine transport and the carnitine cycle**					
3.1.1. Carnitine palmitoyltransferase II (CPTII) deficiency	255110	**CPT2** 1223delCT/c.1891 C>T; p.R631C	Myocytes	Palmitoylcarnitine accumulation	[56]
**3.2. Disorders of mitochondrial fatty acid oxidation**					
3.2.1. Very long-chain acyl-CoA dehydrogenase deficiency	201475	**ACADVL** c. 848T >C (p.Val283Ala) c.1141_1143delGAG (p.Glu381del); c.104delC (p.Pro35Leufs*26)	Cardiomyocytes	Electrophysiological alterations	[57, 58]
3.2.2. Mitochondrial trifunctional protein deficiency	143450	**HADHA** c.1528 G>C; p.E510Q	Retinal pigment epithelium, cardiomyocytes	Lipid accumulation. Inefficient pigmentation. Defect in tight junctions. Defective calcium dynamics and repolarization kinetics. Mitochondrial alterations	[59, 60]
**4. Disorders of energy metabolism**					
**4.1. Disorders of pyruvate metabolism**					

4.1.1. Pyruvate kinase deficiency	266200	**PKLR** c.359 C>T/1168 G>A; IVS9(+1) G>C	Erythrocytes	Energetic imbalance (ATP production impaired)	[61]
**4.2. Mitochondrial respiratory chain disorders**					
4.2.1. Respiratory chain disorders caused by mutations of mtDNA					
4.2.1.1. Large-scale single deletion of mtDNA					
4.2.1.1.1. Pearson Syndrome	557000		Hematopoietic progenitors	Iron granule deposition. Differences in growth, mitochondrial function *vs* control	[62]
4.2.1.1.2. Kearns Sayre Syndrome	530000		Neural progenitor cells, cardiomyocytes	Not phenotype	[62-65]
4.2.1.2. Point mutations of mtDNA					
4.2.1.2.1. Mitochondrial encephalomyopathy lactic acidosis and stroke-like episodes, MELAS	540000	**MT-TL1** m.3243 A>G **MT-TW** m.5541 C>T **mtDNA** m.13513 G>A	Neurons, myoblasts, myocytes, cardiomyocytes, neural progenitor cells, spinal cord organoids, retinal pigment epithelium	Oxidative stress. Enhanced autophagy flux. Low mitophagy. ROS and intracellular calcium increased. Depolarization of mitochondrial membrane potential and reduction of mitochondrial ATP production. Small fragmented mitochondria. Neuronal maturation impaired. Lower synaptic density. Neuronal network activity and synchronicity impaired	[66-76]

4.2.1.2.2. Myoclonic epilepsy associated with ragged red fibres, MERRF	545000	**MT-TK** m.8344 A>G	Inner ear hair cells (HCs), cardiomyocytes, neural progenitor cells	Elevated ROS production. Fragmented mitochondria and impaired functionality. Altered antioxidant gene expression. Failed to acquire mature stereociliary bundles, more single cilia with a shorter length and fewer stereociliary bundle-like protrusions	[77-81]
4.2.1.2.3. Leber Hereditary Optic Neuropathy, LHON	535000	**MT-ND4** m.11778 G>A, m.11778 G>C, m.14484 T>C; m.4160 T>C; m.3460G > A/MT-ND1	Retinal ganglion cells	Defective neurite growth. Oxidative stress. Increased level of apoptosis. Mitochondrial dysfunction	[82-87]
4.2.1.2.4. Maternally inherited Mitochondrial Cardiomyopathy	n/a	**MT-RNR2** m.2336 T>C	Cardiomyocytes	Mitochondrial dysfunction and ultrastructure defects. ATP/ADP ratio and mitochondrial membrane potential reduced. Abnormal Ca^{2+} homeostasis	[88]
4.2.2. Respiratory chain disorders caused by mutations of nuclear DNA					
4.2.2.1. Mitochondrial DNA Depletion Syndromes					
4.2.2.1.1. Alpers-Huttenlocher Syndrome	203700	**POLG** c.1251-2 A>T	Hepatocyte	Valproic acid (VPA) hepatotoxicity increased, higher VPA sensitivity. Abnormal mitochondrial ultrastructure. Mitochondrial dysfunction	[89]

4.2.2.1.2. Hepatocerebral (*DGUOK*, *MPV17*, *PEO1*)	251880	**DGOUK** DGUOK$^{\Delta 14/\Delta 5}$: p.W166Ter; H167L fsTer213	Hepatocyte	Reduction in mtDNA copy number. Mitochondria with decreased matrix density and abnormal cristae. Decrease in mitochondrial membrane potential and electron transport chain deficiencies. Attenuated capacity for energy production. Increase in ROS and lactate levels	[90]
4.2.2.1.3. Childhood-onset autosomal dominant optic atrophy		**OPA1** c.2496+1 G>T	Retinal ganglion cells (RGCs)	Increased apoptosis. Inefficient differentiation into RGCs	[91-93]
4.2.2.1.3.1. Behr syndrome	210000	**OPA1** c.610+364 G>A/c.1311 A>G	Not performed	Not performed	[91]
4.2.2.1.3.2. Optic atrophy 'plus' phenotype		**OPA1** c.1861 C>T; p.Q621Ter	Not performed	Not performed	[92]
4.2.2.1.4. Mitochondrial Neurogastrointestinal Encephalopathy, MNGIE	603041	**TYMP**	Cerebral organoids	Not performed	[94]
4.2.2.2. Multiple mtDNA Deletion Syndromes					
4.2.2.2.1. Progressive External Ophthalmoplegia Autosomal Dominant (PEOA)					

4.2.2.2.1.1. PEOA1	157640	**POLG**<br>c.2243 G>C; p.W748S	Not performed	Not performed	[95]
4.2.2.3. Leigh Syndrome (LS)	256000	**MT-ATP6**<br>c.8993 T>G; p.L156R<br>**MT-ND5**<br>c.13513 G>A; p.D393N<br>**SURF1**<br>c.530T>G p.(V177G)<br>c.769G>A p.(G257R)	Cardiomyocytes, skeletal muscle cells and neural progenitor cells, neurons, brain organoids	Impaired oxygen consumption and ATP production. Mitochondrial dysfunction. High cytoplasmic calcium concentration. Cardiac differentiation impaired. Compromised neuronal morphogenesis	[70, 96-101]
4.2.2.4. Ubiquinone (CoQ10) deficiency (Non-LS)	607426	**COQ4**<br>c.483 G>C; p.E161D	Skeletal muscle, dopaminergic neuron, motor neuron	Impaired mitochondrial function and metabolic defects. Impaired differentiation into skeletal muscle cells	[102, 103]
**5. Disorders in the metabolism of purines, pyrimidines and nucleotides**					
**5.1. Disorders of purine metabolism**					
5.1.1. Adenosine deaminase deficiency	102700	**ADA**<br>GGG>AGGEx7/<br>GAAGAdelEx10	Not performed	Not performed	[104]
5.1.2. Deoxyguanosine kinase deficiency	251880	**DGUOK**<br>p.W166X/H167fs	Hepatocytes	mtDNA depletion, reduced oxidative phosphorylation and energetic capacity	[90]
5.1.3. Lesch-Nyhan syndrome	308000	**HPRT1**<br>Inv/del, ex6-9; IVS7 + 5 G>A; delEx1; c.151 C>T; c.508 C>T; c.371insTT	Neurons	Impaired neural differentiation. Shorter neurites.	[104-110]
5.1.4. Purine nucleoside phosphorylase deficiency	164050	**PNP**	Neurons	Reduced levels of hypoxanthine	[111]

5.1.5. Mitochondrial ribonucleotide reductase subunit 2 deficiency	604712	**RRM2B**<br>10delEx2	Hepatocytes	mtDNA depletion, reduced oxidative phosphorylation and energetic capacity	[90]
**5.2. Disorders of pyrimidine metabolism**					
5.2.1. Thymidine phosphorylase deficiency	131222	**TYMP**	Cerebral organoids	Not performed	[94]
**5.3. Disorders of nucleotide metabolism**					
5.3.1. Aicardi-Goutières syndrome (AGS)					
5.3.1.1. AGS1	225750	**TREX1**<br>c.260insAG/S88fs*22	Not performed	Not performed	[112, 113]
5.3.1.2. AGS2	610181	**RNASEH2B**<br>c.529 G>A	Not performed	Not performed	[112, 114]
5.3.1.3. AGS5	612952	**SAMHD1**<br>Ex14-15del	Not performed	Not performed	[115]
5.3.1.4. AGS7	615846	**IFIH1**<br>c.2471 G>A	Not performed	Not performed	[112, 116]

6. Disorders of the metabolism of sterols

6.1. Disorders of sterol biosynthesis

6.1.1. Smith-Lemli-Opitz syndrome	270400	**DHCR7**<br>c.964-1 G>C; p.T93M	Neural progenitors, retinal pigmented epithelium cells	7-dehydrocholesterol accumulation and decreased cholesterol levels. Defective phagosome maturation. Accumulation of ubiquitinated proteins. Aberrant neural differentiation	[117, 118]
6.1.2 Antley-Bixler syndrome with disordered steroidogenesis	201750	**POR**<br>c.1370G>A	Not performed	Not performed	[119]

6.2. Disorders of bile acid biosynthesis

6.2.1. Oxysterol 7-alpha-hydroxylase deficiency	613812	**CYP7B1**<br>p.Y275X; p.R486C	Not performed	Not performed	[120, 121]
6.2.2. Cerebrotendinous xanthomatosis	213700	**CYP27A1**<br>c.1183 C>A; p.R395S	Not performed	Not performed	[122]

6.3. Disorders of bile acid metabolism and transport

6.3.1. Bilirubin UDP-glucuronosyltransferase 1 deficiency	218800	**UGT1A1**<br>13bp-del Ex2	Not performed	Not performed	[33]
6.3.2. Progressive familial intrahepatic cholestasis type 2	601847	**ABCB11**<br>c.-24 C>A; c.2417 G>A<br>c.2782 C>A (R928X)<br>c.3268 C>T (R1090X)	Hepatocyte	Abnormal protein localization, defects in bile acids excretion, suppression of *de novo* bile acid synthesis	[123, 124]

**7. Disorders of porphyrin and haem metabolism**					
7.1. Congenital erythropoietic porphyria	263700	**UROS**<br>c.217 T>C<br>c.683 C>T	Hematopoietic progenitors	Not performed	[125]
7.2. X-linked sideroblastic anaemia (XLSA)	300751	**ALAS2**<br>c.1737 T>C (V562A)	Erythrocytes	Presence of ring sideroblasts, abnormal mitochondrial iron deposition	[126]
**8. Disorders of lipid and lipoprotein metabolism**					
**8.1. Inherited hypercholesterolaemias**					
8.1.1. Disorder of low density lipoprotein receptor	143890	**LDLR**<br>c.2108_2114dup (A705fsX14);<br>c.654_656delTGG (G219del);<br>c.901 G>T<br>c.97 C>T	Hepatocytes	Impaired transport from endoplasmic reticulum, LDL uptake deficiency, high ApoB and cholesterol secretion	[33, 127-135]
**8.2. Inherited hypertriglyceridaemias**					
8.2.1.1. Familial lipoprotein lipase deficiency	238600	**LPL**<br>c.928 T>C (C310R)	Not performed	Not performed	[136]
**8.3. Disorders of high density lipoprotein metabolism**					
8.3.1. Tangier disease	205400	**ABCA1**<br>E1005X;<br>S2046R/K531N	Hepatocytes	Impaired cholesterol efflux, loss of HDL formation, enhance trygliceride secretion and ANGPL3 expression	[137]
**8.4. Inherited hypolipidaemias**					

8.4.1.	Familial abetalipoproteinaemia	200100	**MTTP**<br>R46G	Hepatocytes, cardiomyocytes	Absent ApoB protein, excess intracelular cholesterol and triglycerides storage, cardiac sensitization to stress	[138]
8.4.2.	Familial hypobetalipoproteinaemia	144010	**APOB**<br>c.10579 C>T (R3527W)	Not performed	Not performed	[139]
**9.**	**Congenital disorders of glycosylation and other disorders of protein modification**					
9.1.	Disorders of protein N-glycosylation					
9.1.1.	Phosphomannomutase 2 deficiency	601785	**PMM2**<br>R141H/F119L	Hepatocytes	Diminished mannose incorporation into proteins, protein hypoglycosylation. Increased cell surface LDLR expression	[140, 141]
9.1.2.	Glucosyltransferase 1 deficiency	603147	**ALG6**	Hepatocytes	Increased cell surface LDLR expression	[141]
9.2.	Disorders of protein O-glycosylation					
9.2.1.	Multiple exostoses type I	133700	**EXT1**<br>c.1883+1G>T	Not performed	Not performed	[142]
9.2.2.	Fukutin-related protein deficiency	606596	**FKRP**<br>c.826 C>A (L276I)<br>c.1364 C>A (A455D)	Cardiomyocytes, cortical neurons	Abnormal action potentials, reduced expression of channel currents, reduced intracellular Ca^{2+} concentrations. Absence of glycosylation in neurons	[143, 144]

10. Lysosomal disorders

10.1. Mucopolysaccharidoses

10.1.1.	MPS I, Hurler, Scheie disease	252800	**IDUA**<br>IVS5AS-7 G>A/ W402X; Y167X/W402X; H358-T364del<br>c.266 G>A	Neural progenitor cells, hematopoietic progenitors	Accumulation of GAG, enlarged lysosomes, dysregulated autophagy pathway. Migration defects	[145-148]
10.1.2.	MPS II, Hunter disease	309900	**IDS**<br>c.182 C>T (S61F); c.1181-1 G>A; c.1403 G>A (R468Q); c.85C>T; c.208insC	β3-Tubulin^{+} neurons, astrocytes, CNPase^{+} oligodendrocytes	Glial and neuronal GAG accumulation, decreased neuronal self-renewal capacity, structural alterations in Golgi and endoplasmatic reticulum, vacuoles accumulation	[149-156]
10.1.3.	MPS IIIA, Sanfilippo A disease	252900	**SGSH**<br>E447K/R245H	Not performed	Not performed	[157]
10.1.4.	MPS IIIB, Sanfilippo B disease	252920	**NAGLU**<br>c.531+1G.C; R482W; P358L; c.457 G>A	Neural precursors, β3-Tubulin^{+} neurons	HS proteoglycans accumulation, disorganized Golgi structure, increased LAMP1 and GM130	[158-160]
10.1.5.	MPS IIIC, Sanfilippo C disease	252930	**HGSNAT**<br>c.633+1 G>A/ c.1334 T>C (L445P); c.372-2 A>G	MAP2^{+} neurons, astrocytes	GAG accumulation, large vacuoles with an empty-like appearance, impaired neuronal network activity and connectivity. Increased in lysosomes in heparin sulfate	[161-163]
10.1.6.	MPS IVA, Morquio A disease	253000	**GALNS**<br>R61W/WT405del	Not performed	Not performed	[164]

10.1.7. MPS VII, Sly disease	253220	**GUSB**<br>L176F	Neural progenitor cells, β3-Tubulin$^+$ neurons	GAG accumulation, expanded endocytic compartments, accumulation of lipofuscin granules, increased number of autophagosomes, reduced neuronal activity and altered network connectivity	[165, 166]
**10.2. Oligosaccharidoses**					
10.2.1. Sialidosis	256550	**NEU1**<br>A544G/c.667_679 del<br>c.649 G>A/644 T>C;<br>c.1109 A>G;<br>c.1195_12000dup/c.679 G>A<br>G227R and V275A/R347Q	Oligodendrocytes, astrocytes and neurons	Impaired lysosomal and autophagic function. Defects in neural differentiation. Glycolitic impairment. Presynaptic dysfunction. Deregulation of Ca^{2+} dynamics	[167-170]
**10.3. Sphingolipidoses**					
10.3.1. GM1-gangliosidosis	230500	**GLB1**<br>R201C<br>I51T	Neural progenitor cells	Defective GLB1 activity, increased lysosomes, activation of inflammasome. Impaired neurotransmitter release, accumulation of GMS1 ganglioside	[171, 172]
10.3.2. GM2-gangliosidosis 0-variant, Sandhoff disease	268800	**HEXB**<br>16kb-del/ IVS10- 2A>G	Cerebral organoids	GM2 accumulation mainly in β3-Tubulin$^+$ neurons, alterations in neuronal differentiation	[173]
10.3.3. GM2-gangliosidosis B-variant, Tay-Sachs disease	272800	**HEXA**<br>c.1278insTATC;<br>c.1278insTATC/W392X<br>1278insTATC/IVS12+1G>C	Neural progenitor cells, β3-Tubulin$^+$ neurons	Accumulation of lipids in NPC lysosomes, GM2 accumulation. Enlarged lysosomes, increased oxidative stress, decreased exocytotic activity	[174-176]

10.3.4.	Gaucher disease	230800	**GBA**<br>N370S/c.84GG;<br>c.1448 T>C (L483P);<br>c.667 T>C (W223R);<br>L444P;<br>N370S;<br>L444P/G202R;<br>W184R/D409H;<br>IVS2+1 G>A/L444P;<br>L444P/P415R;<br>G325R/C342G<br>P213I	Dopaminergic neurons, β3-Tubulin+ neurons, neural progenitor cells, macrophages, hematopoietic progenitor cells, osteoblasts, astrocytes	Compromised lysosomal protein degradation, accumulation of α-synuclein, aggregation-dependent neurotoxicity, abnormal electrophysiological properties, differentiation defect in neurons, accumulation of glucosylsphingolipids and glucosylceramide in macrophages, delay in red blood cells clearance, increased levels of inflammatory cytokines, elevated levels of chitotriosidase, impaired chemotaxis, reduced production of intracellular ROS. Decreased neural TFEB levels, defective bone matrix protein and mineral deposition, defective Ca^{2+}-dependent exocytosis and homeostasis. Astrocyte reactivity. mTOR hyperactivity	[104, 177-197]
10.3.5.	Krabbe disease	245200	**GALC**<br>c.461 C>A/c.1244 G>A	Not performed	Not performed	[198]
10.3.6.	Metachromatic leukodystrophy	250100	**ARSA**<br>c.465+ 1G>A/<br>c.1223_1231del9;<br>c.465+ 1G>A;<br>P426L;<br>c.459+ 1G>A/ c.1049 A>G<br>c.1178 C>G	Neural progenitor cels, β3-Tubulin+ neurons, astrocytes, oligodendrocytes	Sulfatide accumulation, expansion of the endolysosome system, intracellular ROS production, oligodendroglial loss, disorganized neuronal network	[199-202]

10.3.7.	Fabry disease	301500	**GLA**<br>c.485 G>A;<br>c.658 C>T;<br>G485A;<br>C658T;<br>IVS4+919 G>A;<br>c.708 G>C<br>W287S	Cardiomyoctes, vascular endothelial-like cells	Accumulation of globotriaosylceramide, cellular hypertrophy, up-regulation of IL-18, increased ROS production, decreased energy metabolism, arachidonate 12/15-lipoxygenase upregulation. Altered cardiomyocyte electrophysiology and calcium handling. Dysfunctional angiogenesis. Autophagic flux impairment	[203-214]
10.3.8.	Niemann-Pick disease type A or B	257200	**SMPD1**<br>L302P; P330fs<br>p.L43-A44delLA	Neural progenitor cells	Enlarged multilamellar lysosomes, sphingomyelin accumulation	[215-217]
10.3.9.	Niemann-Pick disease type C1	257220	**NPC1**<br>c.1628 delC /E612D;<br>I1061T/P237S;<br>I1061T;<br>1920 delG/1009 G>A;<br>1920 delG;<br>c.1180 T>C<br>V1023fs/pG992R	Neural progenitor cells, β3-Tubulin^{+} neurons, hepatocytes, astrocytes	Lysosomal cholesterol accumulation, enlarged lysosomes, impaired cholesterol trafficking to ER, block in autophagic flux, abnormal VEGF levels and sphingolipid metabolism, increased gene expression of genes involved in neural calcium signaling, disruption of genes involved in neural WNT signaling, glycosphingolipid GM2 accumulation in neurons, increased number of reactive astrocytes, increased activation of the necroptotic pathway	[218-231]
10.3.10.	Niemann-Pick disease type C2	607625	**NPC2**<br>c.58 G>T/c.140 G>T	Neurons and Glia cells	Cholesterol and sphingolipids accumulation.	[232, 233]

**10.4.**	**Ceroid lipfuscinoses, neuronal (CLN)**					
10.4.1.	CLN1, Santavuori-Haltia disease	256730	**PPT1**<br>Y247H/M1I	Neural progenitor cells	Enlarged lysosomes, accumulation of lipids	[234]
10.4.2.	CLN2, Jansky-Bielschowsky disease	204500	**TPP1**<br>R127X/R208X;<br>R127Q/IVS5-1 G>C	Neural progenitor cells	Enlarged lysosomes, accumulation of lipids, accumulation of subunit C of mitocondrial ATP synthase	[234, 235]
10.4.3.	CLN3, Batten Spielmeyer-Vogt disease	204200	**CLN3**<br>1.02-kb del Ex 7-8;<br>c.1056+3 A>C/ c.1247 A>G;<br>1.02-kb del/<br>L101del3CTC	Neural progenitor cells, β3-Tubulin$^+$ neurons, retinal cells, brain microvascular endothelial cells	Cytoplasmic vacuolations, accumulation of subunit C of mitocondrial ATP synthase, bigger autophagic vacuoles, reduced multivesicular bodies, defect in late-stage autophagosome maturation, abnormal calcium handling. Mitochondrial dysfunction, impaired barrier function	[235-238]
10.4.4.	CLN5 Finnish variant	256731	**CLN5**<br>c.1175_1176delAT<br>(Y392X)	Neural progenitors cells	Enlarged lysosomes, endoplasmic reticulum, accumulation of subunit C of the mitochondrial ATP synthase, disturbed sphingolipid transport	[239]
**10.5.**	**Lysosomal export disorders**					
10.5.1.	Cystinosis	219800	**CTNS**<br>57kb del/L158P	Kidney organoids	Elevated cystine levels, defective autophagy, increased apoptosis	[240]

10.6. Other lysosomal disorders

10.6.1.	Wolman/cholesterol ester storage disease	278000	**LIPA**<br>c.594dupT(A199Cfs*13)/ c.796 G>T(p.G266X)	Neural progenitor cells, hepatocyte-like organoids	Accumulation of neutral lipids, increased lysosomal content. Steatosis after oleic acid exposure, increased the fibrosis P3NP biomarker, increased stiffness and ROS production	[241, 242]
10.6.2.	Pompe disease, GSD type II	232300	**GAA**<br>del ex18/del ex18;<br>c.1935 C>A;<br>c.1935 C>A/c.2040 +1 G>T;<br>p.D645E/c.1935 C>A;<br>del Ex18/del525T;<br>c.1062 C>G;<br>p.Y354X/c.1935 C>A;<br>IVS1-13 T>G/ del Ex18;<br>1441delT/2237 G>A;<br>c.796 C>T/c.1316 T>A;<br>IVS1-13 T>G/ del525T;<br>IVS1-13 T>G/ c.923 A>T c.2560C > T (p.R854X); c.1822C>T, p.R608X; c.2662G>T, p.E888X	Cardiomyocytes, skeletal muscle cells, neural progenitors cells, neurons, hepatocytes	Increased lysosomal glycogen accumulation, mitochondrial dysfunction, multiple ultrastructure aberrances, large glycogen-containing vacuoles, glycan processing abnormality, increased oxidative stress, suppressed mTORC1 activation	[36-51]
10.6.3.	Danon disease	300257	**LAMP2**<br>c.129–130 insAT; IVS-1 c.64+1 G>A;<br>c.183_184insA;<br>c.520 C>T;<br>IVS6+1_4delGTGA<br>c.467 T>G;<br>c.247 C>T	Cardiomyocytes, $Map2^+$ neurons	Defect in autophagic flux, cellular hypertrophy, abnormal calcium handling, excessive mitochondrial oxidative stress, accumulation of $LC3^+$ autophagosomes, oxidative stress-induced apoptosis, impaired autophagy of depolarized mitochondria, mitochondrial dysfunction, defect in autophagosome-lysosome fusion	[243-250]

10.6.4. Hermansky-Pudlak Syndrome	203300	**HPS1**<br>c.1472_1487dup16<br>**AP3B1**<br>R509X/E659X	Melanocytes, alveolar type 2 cells	Reduced melanosomes and pigmentation, altered distribution, enlargement, and impaired secretion of lamellar bodies in alveolar cells	[251-254]

11. Peroxisomal disorders

11.1. Disorders of peroxisome biogenesis

11.1.1. Zellweger spectrum disorder	214100	**PEX1**<br>I700fs/G973fs; G843D<br>**PEX10**<br>L113fs; L297P<br>**PEX12**<br>S320F<br>**PEX26**<br>R98W	Neural progenitor cells, Tuj1+ neurons, oligodendrocyte precursor cells, hepatocytes	Poorly branched oligodendrocytes, reduced sVLCFA and phosphatidylethanolamine plasmalogen levels in iPSC, peroxisome assembly defect	[255]

11.2. Disorders of peroxisomal alpha-, beta and omega-oxidation

11.2.1. X-linked adrenoleukodystrophy	300100	**ABCD1**<br>c. 253_254insC;<br>c.1847 C>T (A616V);<br>c.1534 G>A;<br>c.1968_1970delCAT;<br>c.1661 G>A;<br>c.1240-1253del6ins;<br>c.2013insA	Oligodendrocytes, neurons, astrocytes, brain microvascular endothelial cells	Abnormal VLCFA accumulation, increased *ELOVL1* expression and proinflammatory cytokines, *CH25H* overexpression, 25-hydroxycholesterol (25-HC)-dependent NLRP3 inflammasome activation, defective barrier function	[256-264]

**11.3. Other peroxisomal disorders**					
11.3.1. Primary hyperoxaluria type I	259900	**AGXT**<br>I244T<br>p.G170R/R122X	Not performed	AGT protein aggregation	[29-32]
**12. Disorders of neurotransmitter metabolism**					
**12.1. Disorders in the metabolism of biogenic amines**					
12.1.1. Tyrosine hydroxylase deficiency	191290	**TH**<br>R129*/R231P	Not performed	Not performed	[265]
12.1.2. Aromatic L-amino acid decarboxylase (AADC) deficiency	608643	**DDC**<br>c.1039C>G, p.R347G<br>c.19C>T,<br>p.Aeg7*/c.229G>C,<br>p.C100S	Dopaminergic neurons	Dysregulated dopamine metabolism, alterations in synaptic maturation and neuronal electrical properties	[266]
**13. Disorders in the metabolism of vitamins and (non-protein) cofactors**					
**13.1. Disorders of cobalamin absorption, transport and metabolism**					
13.1.1. Defect in adenosylcobalamin synthesis-cbl B	251110	**MMAB**<br>I96T/S174Cfs*23	Not performed	Not performed	[267]
**13.2. Disorders of pterin metabolism**					
13.2.1. Guanosine 5 triphosphate cyclohydrolase I deficiency	233910	**GCH1**<br>L79_S80del	Not performed	Not performed	[268]
13.2.2. 6-Pyruvoyl-tetrahydropterin synthase deficiency	261640	**PTS**<br>c.243 G>A /c.259 C>T	Dopaminergic neurons	Reduction in BH4 amount, TH protein level and extracellular dopamine	[269]
13.2.3. Quinoid dihydropteridine reductase deficiency	261630	**QDPR**<br>c.52 G>T/c.176 C>A	Dopaminergic neurons	Reduced TH protein and extracellular dopamine level, increase of dihydrobiopterin	[269]

**13.3. Other disorders of vitamins and cofactors**					
13.3.1. Pantothenate kinases deficiency	234200	**PANK2**<br>Y190X;<br>F419fsX472;<br>R481Lfs/G521R;<br>IVS4-1 G>T/M437T;<br>R278L/L564P;<br>c.1259delG	Glutamatergic neurons, cortical neurons	Defects in membrane excitability, premature death, increased ROS production, increased mitochondrial membrane potential. Altered iron content and mitochondrial aggregates, altered calcium homeostasis	[270-272]
**14. Disorders in the metabolism of trace elements and metals**					
**14.1. Disorder of copper metabolism**					
14.1.1. Menkes syndrome	309400	**ATP7A**<br>c4005 + 5G>A;<br>c121-930_2626/488del<br>M1311V	Neural progenitor cells, mesenchymal stem cells, osteoblast, motor neurons	Aberrant switch of E-cadherin to N-cadherin, impaired neural rosette formation, impaired osteogenesis. Protein deslocalization in motor neurons, copper accumulation, decreased dendritic complexity and survival.	[273-275]
14.1.2. Wilson disease	277900	**ATP7B**<br>R778L;<br>H1069Q/E1064A;<br>M769V; P992L;<br>c.180-181del	Hepatocytes, neural progenitor cell, osteoblasts	Abnormal cytoplasmic localization of mutated ATP7B, copper-export defect. Lower osteogenic activity	[276-288]
**14.2. Disorder of zinc metabolism**					
14.2.1. Acrodermatitis enteropathica	201100	**ZIP4**<br>192+19 G>A/P200L	Enterocytes	Impaired zinc uptake	[289]
**14.3. Disorder of magnesium metabolism**					
14.3.1. Hypomagnesaemia type 5, renal with ocular involvement	248190	**CLDN19**<br>G20D	Retinal progenitor cells	Altered retinal neurogenesis and maturation in culture	[290]
14.3.2. Gitelman syndrome	263800	**SLC12A3**<br>c.46-47del/c.2963 T>C	Not performed	Not performed	[291]

15. Disorders and variants in the metabolism of xenobiotics

Supplementary references

1. Yang, X., et al., *Generation of an induced pluripotent stem cell line (SDQLCHi009-A) from a patient with 47,XXY and ornithine transcarbamylase deficiency carrying a hemizygote mutation in OTC.* Stem Cell Res, 2020. **43**: p. 101704.
2. Zabulica, M., et al., *Gene Editing Correction of a Urea Cycle Defect in Organoid Stem Cell Derived Hepatocyte-like Cells.* Int J Mol Sci, 2021. **22**(3).
3. Yoshitoshi-Uebayashi, E.Y., et al., *Modelling urea-cycle disorder citrullinemia type 1 with disease-specific iPSCs.* Biochem Biophys Res Commun, 2017. **486**(3): p. 613-619.
4. Akbari, S., et al., *Robust, Long-Term Culture of Endoderm-Derived Hepatic Organoids for Disease Modeling.* Stem Cell Reports, 2019. **13**(4): p. 627-641.
5. Kho, J., et al., *Argininosuccinate Lyase Deficiency Causes an Endothelial-Dependent Form of Hypertension.* Am J Hum Genet, 2018. **103**(2): p. 276-287.
6. Kim, Y., et al., *Malfunction in Mitochondrial beta-Oxidation Contributes to Lipid Accumulation in Hepatocyte-Like Cells Derived from Citrin Deficiency-Induced Pluripotent Stem Cells.* Stem Cells Dev, 2016. **25**(8): p. 636-47.
7. Alonso-Barroso, E., et al., *Generation and characterization of a human iPSC line from a patient with propionic acidemia due to defects in the PCCA gene.* Stem Cell Res, 2017. **23**: p. 173-177.
8. Alonso-Barroso, E., et al., *Cardiomyocytes Derived from Induced Pluripotent Stem Cells as a Disease Model for Propionic Acidemia.* Int J Mol Sci, 2021. **22**(3).
9. Lopez-Marquez, A., et al., *Generation and characterization of a human iPSC line (UAMi004-A) from a patient with propionic acidemia due to defects in the PCCB gene.* Stem Cell Res, 2019. **38**: p. 101469.
10. Pournasr, B. and S.A. Duncan, *Generation of isogenic Propionyl-CoA carboxylase beta subunit (PCCB) deficient induced pluripotent stem cell lines.* Stem Cell Res, 2020. **48**: p. 101953.
11. Dudek, J., et al., *Cardiolipin deficiency affects respiratory chain function and organization in an induced pluripotent stem cell model of Barth syndrome.* Stem Cell Res, 2013. **11**(2): p. 806-19.
12. Wang, G., et al., *Modeling the mitochondrial cardiomyopathy of Barth syndrome with induced pluripotent stem cell and heart-on-chip technologies.* Nat Med, 2014. **20**(6): p. 616-23.
13. Chowdhury, A., et al., *Defective Mitochondrial Cardiolipin Remodeling Dampens HIF-1alpha Expression in Hypoxia.* Cell Rep, 2018. **25**(3): p. 561-570 e6.
14. Dudek, J., et al., *Cardiac-specific succinate dehydrogenase deficiency in Barth syndrome.* EMBO Mol Med, 2016. **8**(2): p. 139-54.
15. Fatica, E.M., et al., *Barth Syndrome: Exploring Cardiac Metabolism with Induced Pluripotent Stem Cell-Derived Cardiomyocytes.* Metabolites, 2019. **9**(12).
16. Guo, X., et al., *Generation of urine-derived iPS cell line via a non-integrative method from a Barth syndrome patient with TAZ gene mutation.* Stem Cell Res, 2020. **47**: p. 101886.
17. Rohani, L., et al., *Reversible Mitochondrial Fragmentation in iPSC-Derived Cardiomyocytes From Children With DCMA, a Mitochondrial Cardiomyopathy.* Can J Cardiol, 2020. **36**(4): p. 554-563.
18. Feng, L., et al., *Cell-Based Therapy for Canavan Disease Using Human iPSC-Derived NPCs and OPCs.* Adv Sci (Weinh), 2020. **7**(23): p. 2002155.
19. Zhang, H., et al., *An integration-free iPSC line (SDQLCHi013-A) derived from a patient with maple syrup urine disease carrying compound heterozygote mutations in BCKDHA gene.* Stem Cell Res, 2019. **41**: p. 101585.
20. Li, Y., et al., *An induced pluripotent stem cell line (SDQLCHi006-A) derived from a patient with maple syrup urine disease type Ib carrying compound heterozygous mutations of p.R168C and p.T322I in BCKDHB gene.* Stem Cell Res, 2019. **40**: p. 101579.
21. Xu, T., et al., *Generation of integration-free induced pluripotent stem cell line (NJMUi001-A) from a phenylketonuria patient.* Stem Cell Res, 2017. **25**: p. 179-182.
22. Howden, S.E., et al., *Genetic correction and analysis of induced pluripotent stem cells from a patient with gyrate atrophy.* Proc Natl Acad Sci U S A, 2011. **108**(16): p. 6537-42.
23. Meyer, J.S., et al., *Optic vesicle-like structures derived from human pluripotent stem cells facilitate a customized approach to retinal disease treatment.* Stem Cells, 2011. **29**(8): p. 1206-18.

24. Arribas-Carreira, L., et al., *Generation and characterization of a human iPSC line (UAMi005-A) from a patient with nonketotic hyperglycinemia due to mutations in the GLDC gene.* Stem Cell Res, 2019. **39**: p. 101503.
25. Hsieh, W.C., et al., *Kidney-differentiated cells derived from Lowe Syndrome patient's iPSCs show ciliogenesis defects and Six2 retention at the Golgi complex.* PLoS One, 2018. **13**(2): p. e0192635.
26. Barnes, J., et al., *Modeling the neuropsychiatric manifestations of Lowe syndrome using induced pluripotent stem cells: defective F-actin polymerization and WAVE-1 expression in neuronal cells.* Mol Autism, 2018. **9**: p. 44.
27. Liu, H., et al., *Transcriptome analysis of neural progenitor cells derived from Lowe syndrome induced pluripotent stem cells: identification of candidate genes for the neurodevelopmental and eye manifestations.* J Neurodev Disord, 2020. **12**(1): p. 14.
28. Qian, R., et al., *Establishment of a human induced pluripotent stem cell line (WMUi031-A) from a Lowe syndrome patient carrying a OCRL gene mutation (c.2626dupA).* Stem Cell Res, 2021. **53**: p. 102294.
29. Zapata-Linares, N., et al., *Generation and characterization of human iPSC lines derived from a Primary Hyperoxaluria Type I patient with p.I244T mutation.* Stem Cell Res, 2016. **16**(1): p. 116-9.
30. Martinez-Turrillas, R., et al., *Generation of an induced pluripotent stem cell line (CIMAi001-A) from a compound heterozygous Primary Hyperoxaluria Type I (PH1) patient carrying p.G170R and p.R122* mutations in the AGXT gene.* Stem Cell Res, 2019. **41**: p. 101626.
31. Esteve, J., et al., *Targeted gene therapy in human-induced pluripotent stem cells from a patient with primary hyperoxaluria type 1 using CRISPR/Cas9 technology.* Biochem Biophys Res Commun, 2019. **517**(4): p. 677-683.
32. Esteve, J., et al., *Generation of induced pluripotent stem cells-derived hepatocyte-like cells for ex vivo gene therapy of primary hyperoxaluria type 1.* Stem Cell Res, 2019. **38**: p. 101467.
33. Rashid, S.T., et al., *Modeling inherited metabolic disorders of the liver using human induced pluripotent stem cells.* J Clin Invest, 2010. **120**(9): p. 3127-36.
34. Katagami, Y., et al., *Generation of a human induced pluripotent stem cell line, BRCi009-A, derived from a patient with glycogen storage disease type 1a.* Stem Cell Res, 2020. **49**: p. 102095.
35. Satoh, D., et al., *Establishment and directed differentiation of induced pluripotent stem cells from glycogen storage disease type Ib patient.* Genes Cells, 2013. **18**(12): p. 1053-69.
36. Huang, H.P., et al., *Human Pompe disease-induced pluripotent stem cells for pathogenesis modeling, drug testing and disease marker identification.* Hum Mol Genet, 2011. **20**(24): p. 4851-64.
37. Higuchi, T., et al., *The generation of induced pluripotent stem cells (iPSCs) from patients with infantile and late-onset types of Pompe disease and the effects of treatment with acid-alpha-glucosidase in Pompe's iPSCs.* Mol Genet Metab, 2014. **112**(1): p. 44-8.
38. Raval, K.K., et al., *Pompe disease results in a Golgi-based glycosylation deficit in human induced pluripotent stem cell-derived cardiomyocytes.* J Biol Chem, 2015. **290**(5): p. 3121-36.
39. Sato, Y., et al., *Disease modeling and lentiviral gene transfer in patient-specific induced pluripotent stem cells from late-onset Pompe disease patient.* Mol Ther Methods Clin Dev, 2015. **2**: p. 15023.
40. Sato, Y., et al., *TFEB overexpression promotes glycogen clearance of Pompe disease iPSC-derived skeletal muscle.* Mol Ther Methods Clin Dev, 2016. **3**: p. 16054.
41. Sato, Y., et al., *Metabolomic Profiling of Pompe Disease-Induced Pluripotent Stem Cell-Derived Cardiomyocytes Reveals That Oxidative Stress Is Associated with Cardiac and Skeletal Muscle Pathology.* Stem Cells Transl Med, 2017. **6**(1): p. 31-39.
42. van der Wal, E., et al., *GAA Deficiency in Pompe Disease Is Alleviated by Exon Inclusion in iPSC-Derived Skeletal Muscle Cells.* Mol Ther Nucleic Acids, 2017. **7**: p. 101-115.
43. van der Wal, E., et al., *Large-Scale Expansion of Human iPSC-Derived Skeletal Muscle Cells for Disease Modeling and Cell-Based Therapeutic Strategies.* Stem Cell Reports, 2018. **10**(6): p. 1975-1990.
44. Yoshida, T., et al., *A Skeletal Muscle Model of Infantile-onset Pompe Disease with Patient-specific iPS Cells.* Sci Rep, 2017. **7**(1): p. 13473.

45. Zhang, Y., et al., *Generation of induced pluripotent stem cells (iPSCs) from an infant with Pompe disease carrying with compound mutations of R608X and E888X in GAA gene.* Stem Cell Res, 2019. **41**: p. 101621.
46. Cheng, Y.S., et al., *A human induced pluripotent stem cell line (TRNDi007-B) from an infantile onset Pompe patient carrying p.R854X mutation in the GAA gene.* Stem Cell Res, 2019. **37**: p. 101435.
47. Cheng, Y.S., et al., *Modeling CNS Involvement in Pompe Disease Using Neural Stem Cells Generated from Patient-Derived Induced Pluripotent Stem Cells.* Cells, 2020. **10**(1).
48. Huang, H.P., et al., *Using human Pompe disease-induced pluripotent stem cell-derived neural cells to identify compounds with therapeutic potential.* Hum Mol Genet, 2019. **28**(23): p. 3880-3894.
49. Yoshida, T., et al., *A Liver Model of Infantile-Onset Pompe Disease Using Patient-Specific Induced Pluripotent Stem Cells.* Front Cell Dev Biol, 2019. **7**: p. 316.
50. Jiwlawat, N., et al., *Micropatterned substrates with physiological stiffness promote cell maturation and Pompe disease phenotype in human induced pluripotent stem cell-derived skeletal myocytes.* Biotechnol Bioeng, 2019. **116**(9): p. 2377-2392.
51. Carlson-Stevermer, J., et al., *Design of efficacious somatic cell genome editing strategies for recessive and polygenic diseases.* Nat Commun, 2020. **11**(1): p. 6277.
52. Ortuno-Costela, M.D.C., et al., *Establishment of a human iPSC line (IISHDOi001-A) from a patient with McArdle disease.* Stem Cell Res, 2017. **23**: p. 188-192.
53. Zhan, Y., et al., *Establishment of a PRKAG2 cardiac syndrome disease model and mechanism study using human induced pluripotent stem cells.* J Mol Cell Cardiol, 2018. **117**: p. 49-61.
54. Ben Jehuda, R., et al., *CRISPR correction of the PRKAG2 gene mutation in the patient's induced pluripotent stem cell-derived cardiomyocytes eliminates electrophysiological and structural abnormalities.* Heart Rhythm, 2018. **15**(2): p. 267-276.
55. Hinson, J.T., et al., *Integrative Analysis of PRKAG2 Cardiomyopathy iPS and Microtissue Models Identifies AMPK as a Regulator of Metabolism, Survival, and Fibrosis.* Cell Rep, 2016. **17**(12): p. 3292-3304.
56. Yasuno, T., et al., *Functional analysis of iPSC-derived myocytes from a patient with carnitine palmitoyltransferase II deficiency.* Biochem Biophys Res Commun, 2014. **448**(2): p. 175-81.
57. Knottnerus, S.J.G., et al., *Electrophysiological Abnormalities in VLCAD Deficient hiPSC-Cardiomyocytes Can Be Improved by Lowering Accumulation of Fatty Acid Oxidation Intermediates.* Int J Mol Sci, 2020. **21**(7).
58. Verkerk, A.O., et al., *Electrophysiological Abnormalities in VLCAD Deficient hiPSC-Cardiomyocytes Do not Improve with Carnitine Supplementation.* Front Pharmacol, 2020. **11**: p. 616834.
59. Polinati, P.P., et al., *Patient-Specific Induced Pluripotent Stem Cell-Derived RPE Cells: Understanding the Pathogenesis of Retinopathy in Long-Chain 3-Hydroxyacyl-CoA Dehydrogenase Deficiency.* Invest Ophthalmol Vis Sci, 2015. **56**(5): p. 3371-82.
60. Miklas, J.W., et al., *TFPa/HADHA is required for fatty acid beta-oxidation and cardiolipin re-modeling in human cardiomyocytes.* Nat Commun, 2019. **10**(1): p. 4671.
61. Garate, Z., et al., *Generation of a High Number of Healthy Erythroid Cells from Gene-Edited Pyruvate Kinase Deficiency Patient-Specific Induced Pluripotent Stem Cells.* Stem Cell Reports, 2015. **5**(6): p. 1053-1066.
62. Cherry, A.B., et al., *Induced pluripotent stem cells with a mitochondrial DNA deletion.* Stem Cells, 2013. **31**(7): p. 1287-97.
63. Lester Sequiera, G., et al., *Generation and Evaluation of Isogenic iPSC as a Source of Cell Replacement Therapies in Patients with Kearns Sayre Syndrome.* Cells, 2021. **10**(3).
64. Srivastava, A., et al., *Human induced pluripotent stem cell (hiPSC) line UOMi006-A derived from PBMCs of a patient with Kearns-Sayre syndrome.* Stem Cell Res, 2021. **53**: p. 102355.
65. Srivastava, A., et al., *Generation of human induced pluripotent stem cell (hiPSC) line UOMi005-A from PBMCs of a patient with Kearns-Sayre syndrome.* Stem Cell Res, 2021. **53**: p. 102283.
66. Hatakeyama, H., et al., *Molecular pathomechanisms and cell-type-specific disease phenotypes of MELAS caused by mutant mitochondrial tRNA(Trp).* Acta Neuropathol Commun, 2015. **3**: p. 52.
67. Kodaira, M., et al., *Impaired respiratory function in MELAS-induced pluripotent stem cells with high heteroplasmy levels.* FEBS Open Bio, 2015. **5**: p. 219-25.

68. Folmes, C.D., et al., *Disease-causing mitochondrial heteroplasmy segregated within induced pluripotent stem cell clones derived from a patient with MELAS.* Stem Cells, 2013. **31**(7): p. 1298-308.
69. Hamalainen, R.H., et al., *Tissue- and cell-type-specific manifestations of heteroplasmic mtDNA 3243A>G mutation in human induced pluripotent stem cell-derived disease model.* Proc Natl Acad Sci U S A, 2013. **110**(38): p. E3622-30.
70. Ma, H., et al., *Metabolic rescue in pluripotent cells from patients with mtDNA disease.* Nature, 2015. **524**(7564): p. 234-8.
71. Lin, D.S., et al., *Oxidative Insults and Mitochondrial DNA Mutation Promote Enhanced Autophagy and Mitophagy Compromising Cell Viability in Pluripotent Cell Model of Mitochondrial Disease.* Cells, 2019. **8**(1).
72. Winanto, et al., *Organoid cultures of MELAS neural cells reveal hyperactive Notch signaling that impacts neurodevelopment.* Cell Death Dis, 2020. **11**(3): p. 182.
73. Klein Gunnewiek, T.M., et al., *m.3243A > G-Induced Mitochondrial Dysfunction Impairs Human Neuronal Development and Reduces Neuronal Network Activity and Synchronicity.* Cell Rep, 2020. **31**(3): p. 107538.
74. Sequiera, G.L., C. Rockman-Greenberg, and S. Dhingra, *Establishment of variant free-iPSC (UOMi003-A) line from patient with mitochondrial encephalopathy, lactic acidosis, and stroke-like episodes.* Stem Cell Res, 2020. **48**: p. 101935.
75. Homma, K., et al., *Taurine rescues mitochondria-related metabolic impairments in the patient-derived induced pluripotent stem cells and epithelial-mesenchymal transition in the retinal pigment epithelium.* Redox Biol, 2021. **41**: p. 101921.
76. Yahata, N., H. Boda, and R. Hata, *Elimination of Mutant mtDNA by an Optimized mpTALEN Restores Differentiation Capacities of Heteroplasmic MELAS-iPSCs.* Mol Ther Methods Clin Dev, 2021. **20**: p. 54-68.
77. Chou, S.J., et al., *Generation of two isogenic human induced pluripotent stem cell lines from a 15year-old female patient with MERRF syndrome and A8344G mutation of mitochondrial DNA.* Stem Cell Res, 2018. **30**: p. 201-205.
78. Chen, Y.C., et al., *ATOH1/RFX1/RFX3 transcription factors facilitate the differentiation and characterisation of inner ear hair cell-like cells from patient-specific induced pluripotent stem cells harbouring A8344G mutation of mitochondrial DNA.* Cell Death Dis, 2018. **9**(4): p. 437.
79. Wu, Y.T., et al., *Generation of an induced pluripotent stem cell (iPSC) line from a 40-year-old patient with the A8344G mutation of mitochondrial DNA and MERRF (myoclonic epilepsy with ragged red fibers) syndrome.* Stem Cell Res, 2018. **27**: p. 10-14.
80. Liang, D., et al., *Generation of MERRF patient-derived induced pluripotent stem cell line iMERRF-C7.* Stem Cell Res, 2016. **17**(3): p. 616-618.
81. Chou, S.J., et al., *Impaired ROS Scavenging System in Human Induced Pluripotent Stem Cells Generated from Patients with MERRF Syndrome.* Sci Rep, 2016. **6**: p. 23661.
82. Lu, H.E., et al., *Generation of patient-specific induced pluripotent stem cells from Leber's hereditary optic neuropathy.* Stem Cell Res, 2018. **28**: p. 56-60.
83. Wu, Y.R., et al., *Bioactivity and gene expression profiles of hiPSC-generated retinal ganglion cells in MT-ND4 mutated Leber's hereditary optic neuropathy.* Exp Cell Res, 2018. **363**(2): p. 299-309.
84. Wong, R.C.B., et al., *Mitochondrial replacement in an iPSC model of Leber's hereditary optic neuropathy.* Aging (Albany NY), 2017. **9**(4): p. 1341-1350.
85. Hung, S.S., et al., *Study of mitochondrial respiratory defects on reprogramming to human induced pluripotent stem cells.* Aging (Albany NY), 2016. **8**(5): p. 945-57.
86. Yang, Y.P., et al., *Glutamate Stimulation Dysregulates AMPA Receptors-Induced Signal Transduction Pathway in Leber's Inherited Optic Neuropathy Patient-Specific hiPSC-Derived Retinal Ganglion Cells.* Cells, 2019. **8**(6).
87. Peron, C., et al., *Generation of a human iPSC line, FINCBi001-A, carrying a homoplasmic m.G3460A mutation in MT-ND1 associated with Leber's Hereditary optic Neuropathy (LHON).* Stem Cell Res, 2020. **48**: p. 101939.

88. Li, S., et al., *Mitochondrial Dysfunctions Contribute to Hypertrophic Cardiomyopathy in Patient iPSC-Derived Cardiomyocytes with MT-RNR2 Mutation.* Stem Cell Reports, 2018. **10**(3): p. 808-821.
89. Li, S., et al., *Valproic acid-induced hepatotoxicity in Alpers syndrome is associated with mitochondrial permeability transition pore opening-dependent apoptotic sensitivity in an induced pluripotent stem cell model.* Hepatology, 2015. **61**(5): p. 1730-9.
90. Jing, R., et al., *A Screen Using iPSC-Derived Hepatocytes Reveals NAD(+) as a Potential Treatment for mtDNA Depletion Syndrome.* Cell Rep, 2018. **25**(6): p. 1469-1484 e5.
91. Hauser, S., et al., *Generation of optic atrophy 1 patient-derived induced pluripotent stem cells (iPS-OPA1-BEHR) for disease modeling of complex optic atrophy syndromes (Behr syndrome).* Stem Cell Res, 2016. **17**(2): p. 426-429.
92. Galera-Monge, T., et al., *Generation of a human iPSC line from a patient with an optic atrophy 'plus' phenotype due to a mutation in the OPA1 gene.* Stem Cell Res, 2016. **16**(3): p. 673-6.
93. Chen, J., et al., *Modeling autosomal dominant optic atrophy using induced pluripotent stem cells and identifying potential therapeutic targets.* Stem Cell Res Ther, 2016. **7**: p. 2.
94. Pacitti, D. and B.E. Bax, *The development of an in vitro cerebral organoid model for investigating the pathomolecular mechanisms associated with the central nervous system involvement in Mitochondrial Neurogastrointestinal Encephalomyopathy (MNGIE).* Nucleosides Nucleotides Nucleic Acids, 2018. **37**(11): p. 603-617.
95. Zurita, F., et al., *Generation of a human iPSC line from a patient with a defect of intergenomic communication.* Stem Cell Res, 2016. **16**(1): p. 120-3.
96. Galera-Monge, T., et al., *Generation of a human iPSC line from a patient with Leigh syndrome caused by a mutation in the MT-ATP6 gene.* Stem Cell Res, 2016. **16**(3): p. 766-9.
97. Galera, T., et al., *Generation of a human iPSC line from a patient with Leigh syndrome.* Stem Cell Res, 2016. **16**(1): p. 63-6.
98. Galera-Monge, T., et al., *The mutation m.13513G>A impairs cardiac function, favoring a neuroectoderm commitment, in a mutant-load dependent way.* J Cell Physiol, 2019. **234**(11): p. 19511-19522.
99. Galera-Monge, T., et al., *Mitochondrial Dysfunction and Calcium Dysregulation in Leigh Syndrome Induced Pluripotent Stem Cell Derived Neurons.* Int J Mol Sci, 2020. **21**(9).
100. Grace, H.E., et al., *mRNA Reprogramming of T8993G Leigh's Syndrome Fibroblast Cells to Create Induced Pluripotent Stem Cell Models for Mitochondrial Disorders.* Stem Cells Dev, 2019. **28**(13): p. 846-859.
101. Inak, G., et al., *Defective metabolic programming impairs early neuronal morphogenesis in neural cultures and an organoid model of Leigh syndrome.* Nat Commun, 2021. **12**(1): p. 1929.
102. Romero-Moya, D., et al., *Genetic Rescue of Mitochondrial and Skeletal Muscle Impairment in an Induced Pluripotent Stem Cells Model of Coenzyme Q10 Deficiency.* Stem Cells, 2017. **35**(7): p. 1687-1703.
103. Romero-Moya, D., et al., *Generation, genome edition and characterization of iPSC lines from a patient with coenzyme Q10 deficiency harboring a heterozygous mutation in COQ4 gene.* Stem Cell Res, 2017. **24**: p. 144-147.
104. Park, I.H., et al., *Disease-specific induced pluripotent stem cells.* Cell, 2008. **134**(5): p. 877-86.
105. Frank, S., B.V. Skryabin, and B. Greber, *A modified TALEN-based system for robust generation of knock-out human pluripotent stem cell lines and disease models.* BMC Genomics, 2013. **14**: p. 773.
106. Mekhoubad, S., et al., *Erosion of dosage compensation impacts human iPSC disease modeling.* Cell Stem Cell, 2012. **10**(5): p. 595-609.
107. Mastrangelo, L., et al., *Purinergic signaling in human pluripotent stem cells is regulated by the housekeeping gene encoding hypoxanthine guanine phosphoribosyltransferase.* Proc Natl Acad Sci U S A, 2012. **109**(9): p. 3377-82.
108. Ruillier, V., et al., *Rescuing compounds for Lesch-Nyhan disease identified using stem cell-based phenotypic screening.* JCI Insight, 2020. **5**(4).
109. Paulis, M., et al., *Chromosome Transplantation: A Possible Approach to Treat Human X-linked Disorders.* Mol Ther Methods Clin Dev, 2020. **17**: p. 369-377.

110. Sutcliffe, D.J., et al., *Induced pluripotent stem cells from subjects with Lesch-Nyhan disease.* Sci Rep, 2021. **11**(1): p. 8523.
111. Park, M., et al., *Intracellular Delivery of Human Purine Nucleoside Phosphorylase by Engineered Diphtheria Toxin Rescues Function in Target Cells.* Mol Pharm, 2018. **15**(11): p. 5217-5226.
112. Genova, E., et al., *Biomarkers and Precision Therapy for Primary Immunodeficiencies: An In Vitro Study Based on Induced Pluripotent Stem Cells From Patients.* Clin Pharmacol Ther, 2020. **108**(2): p. 358-367.
113. Ferraro, R.M., et al., *Generation of three iPSC lines from fibroblasts of a patient with Aicardi Goutieres Syndrome mutated in TREX1.* Stem Cell Res, 2019. **41**: p. 101580.
114. Ferraro, R.M., et al., *Establishment of three iPSC lines from fibroblasts of a patient with Aicardi Goutieres syndrome mutated in RNaseH2B.* Stem Cell Res, 2019. **41**: p. 101620.
115. Fuchs, N.V., et al., *Generation of three induced pluripotent cell lines (iPSCs) from an Aicardi-Goutieres syndrome (AGS) patient harboring a deletion in the genomic locus of the sterile alpha motif and HD domain containing protein 1 (SAMHD1).* Stem Cell Res, 2020. **43**: p. 101697.
116. Masneri, S., et al., *Generation of three isogenic induced Pluripotent Stem Cell lines (iPSCs) from fibroblasts of a patient with Aicardi Goutieres Syndrome carrying a c.2471G>A dominant mutation in IFIH1 gene.* Stem Cell Res, 2019. **41**: p. 101623.
117. Francis, K.R., et al., *Modeling Smith-Lemli-Opitz syndrome with induced pluripotent stem cells reveals a causal role for Wnt/beta-catenin defects in neuronal cholesterol synthesis phenotypes.* Nat Med, 2016. **22**(4): p. 388-96.
118. Ramachandra Rao, S., et al., *Compromised phagosome maturation underlies RPE pathology in cell culture and whole animal models of Smith-Lemli-Opitz Syndrome.* Autophagy, 2018. **14**(10): p. 1796-1817.
119. Guo, X., et al., *Establishment of an induced pluripotent stem cell line from a Antley-Bixler Syndrome (ABS) patient with the homozygote mutation p.R457H (c.1370G>A) in POR gene.* Stem Cell Res, 2021. **52**: p. 102261.
120. Hauser, S., et al., *Generation of induced pluripotent stem cells (iPSCs) from a hereditary spastic paraplegia patient carrying a homozygous Y275X mutation in CYP7B1 (SPG5).* Stem Cell Res, 2016. **17**(2): p. 437-440.
121. Hoflinger, P., et al., *Generation of induced pluripotent stem cells (iPSCs) from a hereditary spastic paraplegia patient carrying a homozygous R486C mutation in CYP7B1 (SPG5).* Stem Cell Res, 2016. **17**(2): p. 422-425.
122. Hoflinger, P., et al., *Induced pluripotent stem cells (iPSCs) derived from cerebrotendinous xanthomatosis (CTX) patient's fibroblasts carrying a R395S mutation.* Stem Cell Res, 2016. **17**(2): p. 433-436.
123. Imagawa, K., et al., *Generation of a bile salt export pump deficiency model using patient-specific induced pluripotent stem cell-derived hepatocyte-like cells.* Sci Rep, 2017. **7**: p. 41806.
124. Hayashi, H., et al., *Modeling Human Bile Acid Transport and Synthesis in Stem Cell-Derived Hepatocytes with a Patient-Specific Mutation.* Stem Cell Reports, 2021. **16**(2): p. 309-323.
125. Bedel, A., et al., *Metabolic correction of congenital erythropoietic porphyria with iPSCs free of reprogramming factors.* Am J Hum Genet, 2012. **91**(1): p. 109-21.
126. Hatta, S., et al., *A defined culture method enabling the establishment of ring sideroblasts from induced pluripotent cells of X-linked sideroblastic anemia.* Haematologica, 2018. **103**(5): p. e188-e191.
127. Cayo, M.A., et al., *JD induced pluripotent stem cell-derived hepatocytes faithfully recapitulate the pathophysiology of familial hypercholesterolemia.* Hepatology, 2012. **56**(6): p. 2163-71.
128. Fattahi, F., et al., *Disease-corrected hepatocyte-like cells from familial hypercholesterolemia-induced pluripotent stem cells.* Mol Biotechnol, 2013. **54**(3): p. 863-73.
129. Ramakrishnan, V.M., et al., *Restoration of Physiologically Responsive Low-Density Lipoprotein Receptor-Mediated Endocytosis in Genetically Deficient Induced Pluripotent Stem Cells.* Sci Rep, 2015. **5**: p. 13231.
130. Yang, J., et al., *Generation of Human Liver Chimeric Mice with Hepatocytes from Familial Hypercholesterolemia Induced Pluripotent Stem Cells.* Stem Cell Reports, 2017. **8**(3): p. 605-618.

131. Omer, L., et al., *CRISPR Correction of a Homozygous Low-Density Lipoprotein Receptor Mutation in Familial Hypercholesterolemia Induced Pluripotent Stem Cells.* Hepatol Commun, 2017. **1**(9): p. 886-898.
132. Cayo, M.A., et al., *A Drug Screen using Human iPSC-Derived Hepatocyte-like Cells Reveals Cardiac Glycosides as a Potential Treatment for Hypercholesterolemia.* Cell Stem Cell, 2017. **20**(4): p. 478-489 e5.
133. Okada, H., et al., *Function and Immunogenicity of Gene-corrected iPSC-derived Hepatocyte-Like Cells in Restoring Low Density Lipoprotein Uptake in Homozygous Familial Hypercholesterolemia.* Sci Rep, 2019. **9**(1): p. 4695.
134. Caron, J., et al., *Low-density lipoprotein receptor-deficient hepatocytes differentiated from induced pluripotent stem cells allow familial hypercholesterolemia modeling, CRISPR/Cas-mediated genetic correction, and productive hepatitis C virus infection.* Stem Cell Res Ther, 2019. **10**(1): p. 221.
135. Omer, L., et al., *Familial hypercholesterolemia class II low-density lipoprotein receptor response to statin treatment.* Dis Model Mech, 2020. **13**(4).
136. Sun, X., et al., *Generation of the induced pluripotent stem cell(iPSC) line (AHQUi001-A) from a patient with familial hypertriglyceridemia (FHTG) carrying a heterozygous p.C310R (c.928 T > C) mutation in LPL gene.* Stem Cell Res, 2020. **45**: p. 101802.
137. Bi, X., et al., *ATP-Binding Cassette Transporter A1 Deficiency in Human Induced Pluripotent Stem Cell-Derived Hepatocytes Abrogates HDL Biogenesis and Enhances Triglyceride Secretion.* EBioMedicine, 2017. **18**: p. 139-145.
138. Liu, Y., et al., *Lack of MTTP Activity in Pluripotent Stem Cell-Derived Hepatocytes and Cardiomyocytes Abolishes apoB Secretion and Increases Cell Stress.* Cell Rep, 2017. **19**(7): p. 1456-1466.
139. Bing, S., et al., *Generation of induced pluripotent stem cell GZHMCi002-A from peripheral blood mononuclear cells with APOB mutation.* Stem Cell Res, 2020. **49**: p. 102054.
140. Thiesler, C.T., et al., *Glycomic Characterization of Induced Pluripotent Stem Cells Derived from a Patient Suffering from Phosphomannomutase 2 Congenital Disorder of Glycosylation (PMM2-CDG).* Mol Cell Proteomics, 2016. **15**(4): p. 1435-52.
141. van den Boogert, M.A.W., et al., *N-Glycosylation Defects in Humans Lower Low-Density Lipoprotein Cholesterol Through Increased Low-Density Lipoprotein Receptor Expression.* Circulation, 2019. **140**(4): p. 280-292.
142. Cui, Y., et al., *Generation of an induced pluripotent stem cell line SMBCi010-A from a male with Multiple Osteochondroma carrying a EXT1 mutation.* Stem Cell Res, 2020. **50**: p. 102111.
143. El-Battrawy, I., et al., *Ion Channel Dysfunctions in Dilated Cardiomyopathy in Limb-Girdle Muscular Dystrophy.* Circ Genom Precis Med, 2018. **11**(3): p. e001893.
144. Kim, J., et al., *A new patient-derived iPSC model for dystroglycanopathies validates a compound that increases glycosylation of alpha-dystroglycan.* EMBO Rep, 2019. **20**(11): p. e47967.
145. Swaroop, M., et al., *Patient iPSC-derived neural stem cells exhibit phenotypes in concordance with the clinical severity of mucopolysaccharidosis I.* Hum Mol Genet, 2018. **27**(20): p. 3612-3626.
146. Tolar, J., et al., *Hematopoietic differentiation of induced pluripotent stem cells from patients with mucopolysaccharidosis type I (Hurler syndrome).* Blood, 2011. **117**(3): p. 839-47.
147. Lito, S., et al., *Generation of human induced pluripotent stem cell line UNIGEi001-A from a 2-years old patient with Mucopolysaccharidosis type IH disease.* Stem Cell Res, 2019. **41**: p. 101604.
148. Suga, M., et al., *Generation of a human induced pluripotent stem cell line, BRCi001-A, derived from a patient with mucopolysaccharidosis type I.* Stem Cell Res, 2019. **36**: p. 101406.
149. Reboun, M., et al., *X-Chromosome Inactivation Analysis in Different Cell Types and Induced Pluripotent Stem Cells Elucidates the Disease Mechanism in a Rare Case of Mucopolysaccharidosis Type II in a Female.* Folia Biol (Praha), 2016. **62**(2): p. 82-9.
150. Varga, E., et al., *Generation of Mucopolysaccharidosis type II (MPS II) human induced pluripotent stem cell (iPSC) line from a 1-year-old male with pathogenic IDS mutation.* Stem Cell Res, 2016. **17**(3): p. 482-484.

151. Varga, E., et al., *Generation of Mucopolysaccharidosis type II (MPS II) human induced pluripotent stem cell (iPSC) line from a 7-year-old male with pathogenic IDS mutation.* Stem Cell Res, 2016. **17**(3): p. 463-465.
152. Varga, E., et al., *Generation of Mucopolysaccharidosis type II (MPS II) human induced pluripotent stem cell (iPSC) line from a 3-year-old male with pathogenic IDS mutation.* Stem Cell Res, 2016. **17**(3): p. 479-481.
153. Varga, E., et al., *Generation of human induced pluripotent stem cell (iPSC) line from an unaffected female carrier of Mucopolysaccharidosis type II (MPS II) disorder.* Stem Cell Res, 2016. **17**(3): p. 514-516.
154. Rybova, J., et al., *Neural cells generated from human induced pluripotent stem cells as a model of CNS involvement in mucopolysaccharidosis type II.* J Inherit Metab Dis, 2018. **41**(2): p. 221-229.
155. Kobolak, J., et al., *Modelling the neuropathology of lysosomal storage disorders through disease-specific human induced pluripotent stem cells.* Exp Cell Res, 2019. **380**(2): p. 216-233.
156. Hong, J., et al., *Generation of an induced pluripotent stem cell line (TRNDi008-A) from a Hunter syndrome patient carrying a hemizygous 208insC mutation in the IDS gene.* Stem Cell Res, 2019. **37**: p. 101451.
157. Vallejo, S., et al., *Generation of two induced pluripotent stem cells lines from Mucopolysaccharydosis IIIA patient: IMEDEAi004-A and IMEDEAi004-B.* Stem Cell Res, 2018. **32**: p. 110-114.
158. Vallejo-Diez, S., et al., *Generation of two induced pluripotent stem cells lines from a Mucopolysaccharydosis IIIB (MPSIIIB) patient.* Stem Cell Res, 2018. **33**: p. 180-184.
159. Lemonnier, T., et al., *Modeling neuronal defects associated with a lysosomal disorder using patient-derived induced pluripotent stem cells.* Hum Mol Genet, 2011. **20**(18): p. 3653-66.
160. Huang, W., et al., *An induced pluripotent stem cell line (TRNDi006-A) from a MPS IIIB patient carrying homozygous mutation of p.Glu153Lys in the NAGLU gene.* Stem Cell Res, 2019. **37**: p. 101427.
161. Canals, I., et al., *Activity and High-Order Effective Connectivity Alterations in Sanfilippo C Patient-Specific Neuronal Networks.* Stem Cell Reports, 2015. **5**(4): p. 546-57.
162. Beneto, N., et al., *Generation of two compound heterozygous HGSNAT-mutated lines from healthy induced pluripotent stem cells using CRISPR/Cas9 to model Sanfilippo C syndrome.* Stem Cell Res, 2019. **41**: p. 101616.
163. Beneto, N., et al., *Neuronal and Astrocytic Differentiation from Sanfilippo C Syndrome iPSCs for Disease Modeling and Drug Development.* J Clin Med, 2020. **9**(3).
164. Li, R., et al., *Generation of an induced pluripotent stem cell line (TRNDi005-A) from a Mucopolysaccharidosis Type IVA (MPS IVA) patient carrying compound heterozygous p.R61W and p.WT405del mutations in the GALNS gene.* Stem Cell Res, 2019. **36**: p. 101408.
165. Griffin, T.A., H.C. Anderson, and J.H. Wolfe, *Ex vivo gene therapy using patient iPSC-derived NSCs reverses pathology in the brain of a homologous mouse model.* Stem Cell Reports, 2015. **4**(5): p. 835-46.
166. Bayo-Puxan, N., et al., *Lysosomal and network alterations in human mucopolysaccharidosis type VII iPSC-derived neurons.* Sci Rep, 2018. **8**(1): p. 16644.
167. Liu, S.P., et al., *Generation of novel induced pluripotent stem cell (iPSC) line from a 16-year-old sialidosis patient with NEU-1 gene mutation.* Stem Cell Res, 2018. **28**: p. 39-43.
168. Han, M.J., et al., *Generation of human induced pluripotent stem cells (hIPSCs) from sialidosis types I and II patients with pathogenic neuraminidase 1 mutations.* Stem Cell Res, 2020. **46**: p. 101836.
169. Seol, B., Y.D. Kim, and Y.S. Cho, *Modeling Sialidosis with Neural Precursor Cells Derived from Patient-Derived Induced Pluripotent Stem Cells.* Int J Mol Sci, 2021. **22**(9).
170. Odaka, H., et al., *An iPSC-based neural model of sialidosis uncovers glycolytic impairment-causing presynaptic dysfunction and deregulation of Ca(2+) dynamics.* Neurobiol Dis, 2021. **152**: p. 105279.
171. Son, M.Y., et al., *A novel human model of the neurodegenerative disease GM1 gangliosidosis using induced pluripotent stem cells demonstrates inflammasome activation.* J Pathol, 2015. **237**(1): p. 98-110.

172. Kajihara, R., et al., *Novel Drug Candidates Improve Ganglioside Accumulation and Neural Dysfunction in GM1 Gangliosidosis Models with Autophagy Activation.* Stem Cell Reports, 2020. **14**(5): p. 909-923.
173. Allende, M.L., et al., *Cerebral organoids derived from Sandhoff disease-induced pluripotent stem cells exhibit impaired neurodifferentiation.* J Lipid Res, 2018. **59**(3): p. 550-563.
174. Vu, M., et al., *Neural stem cells for disease modeling and evaluation of therapeutics for Tay-Sachs disease.* Orphanet J Rare Dis, 2018. **13**(1): p. 152.
175. Liu, Z. and R. Zhao, *Generation of HEXA-deficient hiPSCs from fibroblasts of a Tay-Sachs disease patient.* Stem Cell Res, 2016. **17**(2): p. 289-291.
176. Matsushita, K., et al., *Presynaptic Dysfunction in Neurons Derived from Tay-Sachs iPSCs.* Neuroscience, 2019. **414**: p. 128-140.
177. Mazzulli, J.R., et al., *Gaucher disease glucocerebrosidase and alpha-synuclein form a bidirectional pathogenic loop in synucleinopathies.* Cell, 2011. **146**(1): p. 37-52.
178. Nagel, M., et al., *Generation of two iPSC lines derived from two unrelated patients with Gaucher disease.* Stem Cell Res, 2019. **35**: p. 101336.
179. Panicker, L.M., et al., *Induced pluripotent stem cell model recapitulates pathologic hallmarks of Gaucher disease.* Proc Natl Acad Sci U S A, 2012. **109**(44): p. 18054-9.
180. Tiscornia, G., et al., *Neuronopathic Gaucher's disease: induced pluripotent stem cells for disease modelling and testing chaperone activity of small compounds.* Hum Mol Genet, 2013. **22**(4): p. 633-45.
181. Panicker, L.M., et al., *Gaucher iPSC-derived macrophages produce elevated levels of inflammatory mediators and serve as a new platform for therapeutic development.* Stem Cells, 2014. **32**(9): p. 2338-49.
182. Aflaki, E., et al., *Macrophage models of Gaucher disease for evaluating disease pathogenesis and candidate drugs.* Sci Transl Med, 2014. **6**(240): p. 240ra73.
183. Schondorf, D.C., et al., *iPSC-derived neurons from GBA1-associated Parkinson's disease patients show autophagic defects and impaired calcium homeostasis.* Nat Commun, 2014. **5**: p. 4028.
184. Sun, Y., et al., *Properties of neurons derived from induced pluripotent stem cells of Gaucher disease type 2 patient fibroblasts: potential role in neuropathology.* PLoS One, 2015. **10**(3): p. e0118771.
185. Sgambato, J.A., et al., *Gaucher Disease-Induced Pluripotent Stem Cells Display Decreased Erythroid Potential and Aberrant Myelopoiesis.* Stem Cells Transl Med, 2015. **4**(8): p. 878-86.
186. Awad, O., et al., *Altered TFEB-mediated lysosomal biogenesis in Gaucher disease iPSC-derived neuronal cells.* Hum Mol Genet, 2015. **24**(20): p. 5775-88.
187. Aflaki, E., et al., *A New Glucocerebrosidase Chaperone Reduces alpha-Synuclein and Glycolipid Levels in iPSC-Derived Dopaminergic Neurons from Patients with Gaucher Disease and Parkinsonism.* J Neurosci, 2016. **36**(28): p. 7441-52.
188. Awad, O., et al., *Altered Differentiation Potential of Gaucher's Disease iPSC Neuronal Progenitors due to Wnt/beta-Catenin Downregulation.* Stem Cell Reports, 2017. **9**(6): p. 1853-1867.
189. Panicker, L.M., et al., *Gaucher disease iPSC-derived osteoblasts have developmental and lysosomal defects that impair bone matrix deposition.* Hum Mol Genet, 2018. **27**(5): p. 811-822.
190. Kim, M.J., et al., *Acid ceramidase inhibition ameliorates alpha-synuclein accumulation upon loss of GBA1 function.* Hum Mol Genet, 2018. **27**(11): p. 1972-1988.
191. Aflaki, E., et al., *A characterization of Gaucher iPS-derived astrocytes: Potential implications for Parkinson's disease.* Neurobiol Dis, 2020. **134**: p. 104647.
192. Tofoli, F.A., et al., *Generation of three human induced pluripotent stem cell (hiPSC) lines derived from one Gaucher disease patient with Parkinson's disease and two unrelated Parkinson's disease patients with GBA mutations.* Stem Cell Res, 2019. **39**: p. 101519.
193. Duarte, A.J., et al., *Induced pluripotent stem cell line (INSAi001-A) from a Gaucher disease type 3 patient compound heterozygote for mutations in the GBA1 gene.* Stem Cell Res, 2019. **41**: p. 101595.
194. Zhao, Q., et al., *Development of a human iPSC line (SMBCi004-A) from a patient with Gaucher disease.* Stem Cell Res, 2020. **48**: p. 101989.

195. Srikanth, M.P. and R.A. Feldman, *Elevated Dkk1 Mediates Downregulation of the Canonical Wnt Pathway and Lysosomal Loss in an iPSC Model of Neuronopathic Gaucher Disease.* Biomolecules, 2020. **10**(12).
196. Brown, R.A., et al., *mTOR hyperactivity mediates lysosomal dysfunction in Gaucher's disease iPSC-neuronal cells.* Dis Model Mech, 2019. **12**(10).
197. Akiyama, T., et al., *Synthetic mRNA-based differentiation method enables early detection of Parkinson's phenotypes in neurons derived from Gaucher disease-induced pluripotent stem cells.* Stem Cells Transl Med, 2021. **10**(4): p. 572-581.
198. Wang, W., et al., *Generation of a human induced pluripotent stem cell line PUMCi001-A from a patient with Krabbe disease.* Stem Cell Res, 2020. **48**: p. 101937.
199. Doerr, J., et al., *Arylsulfatase A Overexpressing Human iPSC-derived Neural Cells Reduce CNS Sulfatide Storage in a Mouse Model of Metachromatic Leukodystrophy.* Mol Ther, 2015. **23**(9): p. 1519-31.
200. Meneghini, V., et al., *Generation of Human Induced Pluripotent Stem Cell-Derived Bona Fide Neural Stem Cells for Ex Vivo Gene Therapy of Metachromatic Leukodystrophy.* Stem Cells Transl Med, 2017. **6**(2): p. 352-368.
201. Frati, G., et al., *Human iPSC-based models highlight defective glial and neuronal differentiation from neural progenitor cells in metachromatic leukodystrophy.* Cell Death Dis, 2018. **9**(6): p. 698.
202. Kim, K.P., et al., *Generation of a human iPSC line (MPIi007-A) from a patient with Metachromatic leukodystrophy.* Stem Cell Res, 2020. **48**: p. 101993.
203. Kawagoe, S., et al., *Morphological features of iPS cells generated from Fabry disease skin fibroblasts using Sendai virus vector (SeVdp).* Mol Genet Metab, 2013. **109**(4): p. 386-9.
204. Itier, J.M., et al., *Effective clearance of GL-3 in a human iPSC-derived cardiomyocyte model of Fabry disease.* J Inherit Metab Dis, 2014. **37**(6): p. 1013-22.
205. Chien, Y., et al., *Interleukin-18 deteriorates Fabry cardiomyopathy and contributes to the development of left ventricular hypertrophy in Fabry patients with GLA IVS4+919 G>A mutation.* Oncotarget, 2016. **7**(52): p. 87161-87179.
206. Tseng, W.L., et al., *Imbalanced Production of Reactive Oxygen Species and Mitochondrial Antioxidant SOD2 in Fabry Disease-Specific Human Induced Pluripotent Stem Cell-Differentiated Vascular Endothelial Cells.* Cell Transplant, 2017. **26**(3): p. 513-527.
207. Chou, S.J., et al., *Energy utilization of induced pluripotent stem cell-derived cardiomyocyte in Fabry disease.* Int J Cardiol, 2017. **232**: p. 255-263.
208. Chien, Y., et al., *Inhibition of Arachidonate 12/15-Lipoxygenase Improves alpha-Galactosidase Efficacy in iPSC-Derived Cardiomyocytes from Fabry Patients.* Int J Mol Sci, 2018. **19**(5).
209. Kuramoto, Y., et al., *Generation of Fabry cardiomyopathy model for drug screening using induced pluripotent stem cell-derived cardiomyocytes from a female Fabry patient.* J Mol Cell Cardiol, 2018. **121**: p. 256-265.
210. Klein, T., et al., *Generation of the human induced pluripotent stem cell line (UKWNLi001-A) from skin fibroblasts of a woman with Fabry disease carrying the X-chromosomal heterozygous c.708G>C (W236C) missense mutation in exon 5 of the alpha-galactosidase-A gene.* Stem Cell Res, 2018. **31**: p. 222-226.
211. Duarte, A.J., et al., *Induced pluripotent stem cell line (INSAi002-A) from a Fabry Disease patient hemizygote for the rare p.W287X mutation.* Stem Cell Res, 2020. **45**: p. 101794.
212. Birket, M.J., et al., *A Human Stem Cell Model of Fabry Disease Implicates LIMP-2 Accumulation in Cardiomyocyte Pathology.* Stem Cell Reports, 2019. **13**(2): p. 380-393.
213. Do, H.S., et al., *Enhanced thrombospondin-1 causes dysfunction of vascular endothelial cells derived from Fabry disease-induced pluripotent stem cells.* EBioMedicine, 2020. **52**: p. 102633.
214. Song, H.Y., et al., *Reversal of the Inflammatory Responses in Fabry Patient iPSC-Derived Cardiovascular Endothelial Cells by CRISPR/Cas9-Corrected Mutation.* Int J Mol Sci, 2021. **22**(5).
215. Long, Y., et al., *Induced Pluripotent Stem Cells for Disease Modeling and Evaluation of Therapeutics for Niemann-Pick Disease Type A.* Stem Cells Transl Med, 2016. **5**(12): p. 1644-1655.

216. Baskfield, A., et al., *Generation of an induced pluripotent stem cell line (TRNDi004-I) from a Niemann-Pick disease type B patient carrying a heterozygous mutation of p.L43_A44delLA in the SMPD1 gene.* Stem Cell Res, 2019. **37**: p. 101436.
217. Baskfield, A., et al., *An induced pluripotent stem cell line (TRNDi009-C) from a Niemann-Pick disease type A patient carrying a heterozygous p.L302P (c.905 T>C) mutation in the SMPD1 gene.* Stem Cell Res, 2019. **38**: p. 101461.
218. Trilck, M., et al., *Niemann-Pick type C1 patient-specific induced pluripotent stem cells display disease specific hallmarks.* Orphanet J Rare Dis, 2013. **8**: p. 144.
219. Yu, D., et al., *Niemann-Pick Disease Type C: Induced Pluripotent Stem Cell-Derived Neuronal Cells for Modeling Neural Disease and Evaluating Drug Efficacy.* J Biomol Screen, 2014. **19**(8): p. 1164-73.
220. Maetzel, D., et al., *Genetic and chemical correction of cholesterol accumulation and impaired autophagy in hepatic and neural cells derived from Niemann-Pick Type C patient-specific iPS cells.* Stem Cell Reports, 2014. **2**(6): p. 866-80.
221. Lee, H., et al., *Pathological roles of the VEGF/SphK pathway in Niemann-Pick type C neurons.* Nat Commun, 2014. **5**: p. 5514.
222. Soga, M., et al., *HPGCD outperforms HPBCD as a potential treatment for Niemann-Pick disease type C during disease modeling with iPS cells.* Stem Cells, 2015. **33**(4): p. 1075-88.
223. Efthymiou, A.G., et al., *Rescue of an in vitro neuron phenotype identified in Niemann-Pick disease, type C1 induced pluripotent stem cell-derived neurons by modulating the WNT pathway and calcium signaling.* Stem Cells Transl Med, 2015. **4**(3): p. 230-8.
224. Trilck, M., et al., *Diversity of glycosphingolipid GM2 and cholesterol accumulation in NPC1 patient-specific iPSC-derived neurons.* Brain Res, 2017. **1657**: p. 52-61.
225. Cougnoux, A., et al., *Necroptosis in Niemann-Pick disease, type C1: a potential therapeutic target.* Cell Death Dis, 2016. **7**: p. e2147.
226. Rabenstein, M., et al., *Decreased calcium flux in Niemann-Pick type C1 patient-specific iPSC-derived neurons due to higher amount of calcium-impermeable AMPA receptors.* Mol Cell Neurosci, 2017. **83**: p. 27-36.
227. Peter, F., et al., *Activation of PKC triggers rescue of NPC1 patient specific iPSC derived glial cells from gliosis.* Orphanet J Rare Dis, 2017. **12**(1): p. 145.
228. Li, R., et al., *An induced pluripotent stem cell line (TRNDi001-D) from a Niemann-Pick disease type C1 (NPC1) patient carrying a homozygous p. I1061T (c. 3182T>C) mutation in the NPC1 gene.* Stem Cell Res, 2020. **44**: p. 101737.
229. Volkner, C., et al., *Generation of an iPSC line (AKOSi006-A) from fibroblasts of an NPC1 patient, carrying the homozygous mutation p.I1061T (c.3182 T > C) and a control iPSC line (AKOSi007-A) using a non-integrating Sendai virus system.* Stem Cell Res, 2020. **49**: p. 102056.
230. Volkner, C., et al., *Generation of an iPSC line (AKOSi004-A) from fibroblasts of a female adult NPC1 patient, carrying the compound heterozygous mutation p.Val1023Serfs*15/p.Gly992Arg and of an iPSC line (AKOSi005-A) from a female adult control individual.* Stem Cell Res, 2020. **50**: p. 102127.
231. Jurs, A.V., et al., *Oxidative Stress and Alterations in the Antioxidative Defense System in Neuronal Cells Derived from NPC1 Patient-Specific Induced Pluripotent Stem Cells.* Int J Mol Sci, 2020. **21**(20).
232. Volkner, C., et al., *Generation of the Niemann-Pick type C2 patient-derived iPSC line AKOSi001-A.* Stem Cell Res, 2019. **41**: p. 101606.
233. Liedtke, M., et al., *Pathophysiological In Vitro Profile of Neuronal Differentiated Cells Derived from Niemann-Pick Disease Type C2 Patient-Specific iPSCs Carrying the NPC2 Mutations c.58G>T/c.140G>T.* Int J Mol Sci, 2021. **22**(8).
234. Sima, N., et al., *Neural stem cells for disease modeling and evaluation of therapeutics for infantile (CLN1/PPT1) and late infantile (CLN2/TPP1) neuronal ceroid lipofuscinoses.* Orphanet J Rare Dis, 2018. **13**(1): p. 54.
235. Lojewski, X., et al., *Human iPSC models of neuronal ceroid lipofuscinosis capture distinct effects of TPP1 and CLN3 mutations on the endocytic pathway.* Hum Mol Genet, 2014. **23**(8): p. 2005-22.

236. Chandrachud, U., et al., *Unbiased Cell-based Screening in a Neuronal Cell Model of Batten Disease Highlights an Interaction between Ca2+ Homeostasis, Autophagy, and CLN3 Protein Function.* J Biol Chem, 2015. **290**(23): p. 14361-80.
237. Wiley, L.A., et al., *Using Patient-Specific Induced Pluripotent Stem Cells and Wild-Type Mice to Develop a Gene Augmentation-Based Strategy to Treat CLN3-Associated Retinal Degeneration.* Hum Gene Ther, 2016. **27**(10): p. 835-846.
238. Kinarivala, N., et al., *An iPSC-Derived Neuron Model of CLN3 Disease Facilitates Small Molecule Phenotypic Screening.* ACS Pharmacol Transl Sci, 2020. **3**(5): p. 931-947.
239. Uusi-Rauva, K., et al., *Induced Pluripotent Stem Cells Derived from a CLN5 Patient Manifest Phenotypic Characteristics of Neuronal Ceroid Lipofuscinoses.* Int J Mol Sci, 2017. **18**(5).
240. Hollywood, J.A., et al., *Use of Human Induced Pluripotent Stem Cells and Kidney Organoids To Develop a Cysteamine/mTOR Inhibition Combination Therapy for Cystinosis.* J Am Soc Nephrol, 2020. **31**(5): p. 962-982.
241. Aguisanda, F., et al., *Neural stem cells for disease modeling of Wolman disease and evaluation of therapeutics.* Orphanet J Rare Dis, 2017. **12**(1): p. 120.
242. Ouchi, R., et al., *Modeling Steatohepatitis in Humans with Pluripotent Stem Cell-Derived Organoids.* Cell Metab, 2019. **30**(2): p. 374-384 e6.
243. Hashem, S.I., et al., *Brief Report: Oxidative Stress Mediates Cardiomyocyte Apoptosis in a Human Model of Danon Disease and Heart Failure.* Stem Cells, 2015. **33**(7): p. 2343-50.
244. Law, C.Y., et al., *Lysosomal membrane permeabilization is involved in oxidative stress-induced apoptotic cell death in LAMP2-deficient iPSCs-derived cerebral cortical neurons.* Biochem Biophys Rep, 2016. **5**: p. 335-345.
245. Ng, K.M., et al., *Amelioration of X-Linked Related Autophagy Failure in Danon Disease With DNA Methylation Inhibitor.* Circulation, 2016. **134**(18): p. 1373-1389.
246. Hashem, S.I., et al., *Impaired mitophagy facilitates mitochondrial damage in Danon disease.* J Mol Cell Cardiol, 2017. **108**: p. 86-94.
247. Yoshida, S., et al., *Characteristics of induced pluripotent stem cells from clinically divergent female monozygotic twins with Danon disease.* J Mol Cell Cardiol, 2018. **114**: p. 234-242.
248. Chi, C., et al., *LAMP-2B regulates human cardiomyocyte function by mediating autophagosome-lysosome fusion.* Proc Natl Acad Sci U S A, 2019. **116**(2): p. 556-565.
249. Zhu, X., et al., *Generation of induced pluripotent stem cells (NJDTHi001-A) from a Danon disease child with mutation of c.467 T > G in LAMP2 gene.* Stem Cell Res, 2020. **49**: p. 101994.
250. Del Favero, G., et al., *Danon Disease-Associated LAMP-2 Deficiency Drives Metabolic Signature Indicative of Mitochondrial Aging and Fibrosis in Cardiac Tissue and hiPSC-Derived Cardiomyocytes.* J Clin Med, 2020. **9**(8).
251. Mica, Y., et al., *Modeling neural crest induction, melanocyte specification, and disease-related pigmentation defects in hESCs and patient-specific iPSCs.* Cell Rep, 2013. **3**(4): p. 1140-52.
252. Maguire, J.A., et al., *Generation of Hermansky Pudlak syndrome type 2 (HPS2) induced pluripotent stem cells (iPSCs).* Stem Cell Res, 2016. **16**(2): p. 287-9.
253. Maguire, J.A., et al., *Generation of Hermansky-Pudlak Syndrome Type 1 (HPS1) induced pluripotent stem cells (iPSCs).* Stem Cell Res, 2016. **16**(2): p. 233-5.
254. Korogi, Y., et al., *In Vitro Disease Modeling of Hermansky-Pudlak Syndrome Type 2 Using Human Induced Pluripotent Stem Cell-Derived Alveolar Organoids.* Stem Cell Reports, 2019. **12**(3): p. 431-440.
255. Wang, X.M., et al., *Induced pluripotent stem cell models of Zellweger spectrum disorder show impaired peroxisome assembly and cell type-specific lipid abnormalities.* Stem Cell Res Ther, 2015. **6**: p. 158.
256. Jang, J., et al., *Induced pluripotent stem cell models from X-linked adrenoleukodystrophy patients.* Ann Neurol, 2011. **70**(3): p. 402-9.
257. Wang, X.M., et al., *The gene expression profiles of induced pluripotent stem cells from individuals with childhood cerebral adrenoleukodystrophy are consistent with proposed mechanisms of pathogenesis.* Stem Cell Res Ther, 2012. **3**(5): p. 39.

258. Baarine, M., et al., *Functional Characterization of IPSC-Derived Brain Cells as a Model for X-Linked Adrenoleukodystrophy.* PLoS One, 2015. **10**(11): p. e0143238.
259. Jang, J., et al., *25-hydroxycholesterol contributes to cerebral inflammation of X-linked adrenoleukodystrophy through activation of the NLRP3 inflammasome.* Nat Commun, 2016. **7**: p. 13129.
260. Son, D., et al., *Generation of two induced pluripotent stem cell (iPSC) lines from X-linked adrenoleukodystrophy (X-ALD) patients with adrenomyeloneuropathy (AMN).* Stem Cell Res, 2017. **25**: p. 46-49.
261. You, Y.R., et al., *Generation of induced pluripotent stem cell (iPSC) line from a 21-year-old X-linked adrenoleukodystrophy (X-ALD) patient.* Stem Cell Res, 2017. **25**: p. 136-138.
262. Lee, C.A.A., et al., *Modeling and rescue of defective blood-brain barrier function of induced brain microvascular endothelial cells from childhood cerebral adrenoleukodystrophy patients.* Fluids Barriers CNS, 2018. **15**(1): p. 9.
263. Yeon, G.B., et al., *Generation of an induced pluripotent stem cell (iPSC) line from a 42-year-old adult cerebral type X-linked adrenoleukodystrophy (X-ALD) patient.* Stem Cell Res, 2019. **36**: p. 101425.
264. Wang, L., et al., *Generation of an urine-derived induced pluripotent stem cell line from a 6-year old X-linked adrenoleukodystrophy (X-ALD) patient.* Stem Cell Res, 2021. **51**: p. 102170.
265. Jung-Klawitter, S., et al., *Generation of an iPSC line from a patient with tyrosine hydroxylase (TH) deficiency: TH-1 iPSC.* Stem Cell Res, 2016. **17**(3): p. 580-583.
266. Rossignoli, G., et al., *Aromatic l-amino acid decarboxylase deficiency: a patient-derived neuronal model for precision therapies.* Brain, 2021.
267. Richard, E., et al., *Generation and characterization of two human iPSC lines from patients with methylmalonic acidemia cblB type.* Stem Cell Res, 2018. **29**: p. 143-147.
268. Jung-Klawitter, S., et al., *Generation of an iPSC line from a patient with GTP cyclohydrolase 1 (GCH1) deficiency: HDMC0061i-GCH1.* Stem Cell Res, 2017. **20**: p. 38-41.
269. Ishikawa, T., et al., *Genetic and pharmacological correction of aberrant dopamine synthesis using patient iPSCs with BH4 metabolism disorders.* Hum Mol Genet, 2016. **25**(23): p. 5188-5197.
270. Orellana, D.I., et al., *Coenzyme A corrects pathological defects in human neurons of PANK2-associated neurodegeneration.* EMBO Mol Med, 2016. **8**(10): p. 1197-1211.
271. Arber, C., et al., *iPSC-derived neuronal models of PANK2-associated neurodegeneration reveal mitochondrial dysfunction contributing to early disease.* PLoS One, 2017. **12**(9): p. e0184104.
272. Santambrogio, P., et al., *Harmful Iron-Calcium Relationship in Pantothenate kinase Associated Neurodegeneration.* Int J Mol Sci, 2020. **21**(10).
273. Suh, J.H., et al., *Modeling of Menkes disease via human induced pluripotent stem cells.* Biochem Biophys Res Commun, 2014. **444**(3): p. 311-8.
274. Kim, D., et al., *Impaired osteogenesis in Menkes disease-derived induced pluripotent stem cells.* Stem Cell Res Ther, 2015. **6**: p. 160.
275. Bakkar, N., et al., *The M1311V variant of ATP7A is associated with impaired trafficking and copper homeostasis in models of motor neuron disease.* Neurobiol Dis, 2021. **149**: p. 105228.
276. Zhang, S., et al., *Rescue of ATP7B function in hepatocyte-like cells from Wilson's disease induced pluripotent stem cells using gene therapy or the chaperone drug curcumin.* Hum Mol Genet, 2011. **20**(16): p. 3176-87.
277. Yi, F., et al., *Establishment of hepatic and neural differentiation platforms of Wilson's disease specific induced pluripotent stem cells.* Protein Cell, 2012. **3**(11): p. 855-63.
278. Parisi, S., et al., *Characterization of the most frequent ATP7B mutation causing Wilson disease in hepatocytes from patient induced pluripotent stem cells.* Sci Rep, 2018. **8**(1): p. 6247.
279. Liu, J., et al., *A cellular model for Wilson's disease using patient-derived induced pluripotent stem cells revealed aberrant beta-catenin pathway during osteogenesis.* Biochem Biophys Res Commun, 2019. **513**(2): p. 386-391.
280. Zhang, J., et al., *Generation of an integration-free induced pluripotent stem cell (iPSC) line (ZZUNEUi003-A) from a Wilson's disease patient harboring a homozygous R778L mutation in ATP7B gene.* Stem Cell Res, 2019. **41**: p. 101664.

281. Wei, L., et al., *Generation of an integration-free induced pluripotent stem cell (iPSC) line (ZZUNEUi005-A) from a Wilson's disease patient harboring a homozygous Pro992Leu mutation in ATP7B gene.* Stem Cell Res, 2020. **47**: p. 101909.
282. Zhang, J., et al., *Generation of an integration-free induced pluripotent stem cell (iPSC) line (ZZUNEUi004-A) from a Wilson's disease patient harboring a homozygous Pro992Leu mutation in ATP7B gene.* Stem Cell Res, 2020. **44**: p. 101741.
283. Wang, J., et al., *Generation of ZJUi003-A, an induced pluripotent stem cell line from a Wilson's disease patient carrying a c.180_181del mutation in ATP7B gene.* Stem Cell Res, 2020. **46**: p. 101873.
284. Wang, S.H. and X.P. Wang, *Generation of an induced pluripotent stem cell (iPSC) line (THSJTUi001-A) from a Wilson's disease patient harboring a homozygous Arg778Leu mutation in ATP7B gene.* Stem Cell Res, 2020. **49**: p. 102050.
285. Malakhova, A.A., et al., *Generation of two induced pluripotent stem cell lines from peripheral blood mononuclear cells of a patient with Wilson's disease.* Stem Cell Res, 2020. **47**: p. 101922.
286. Petters, J., et al., *Generation of two induced pluripotent stem cell lines from a female adult homozygous for the Wilson disease associated ATP7B variant p.H1069Q (AKOSi008-A) and a healthy control (AKOSi009-A).* Stem Cell Res, 2020. **49**: p. 102079.
287. Petters, J., et al., *Generation of induced pluripotent stem cell lines AKOSi002-A and AKOSi003-A from symptomatic female adults with Wilson disease.* Stem Cell Res, 2020. **43**: p. 101708.
288. Kim, D., et al., *Human Embryonic Stem Cell-Derived Wilson's Disease Model for Screening Drug Efficacy.* Cells, 2020. **9**(4).
289. Sauer, A.K., et al., *Characterization of zinc amino acid complexes for zinc delivery in vitro using Caco-2 cells and enterocytes from hiPSC.* Biometals, 2017. **30**(5): p. 643-661.
290. Wang, S.B., et al., *Disease-associated mutations of claudin-19 disrupt retinal neurogenesis and visual function.* Commun Biol, 2019. **2**: p. 113.
291. Lim, S.W., et al., *Generation of a human induced pluripotent stem cell line (CMCi002-A) from a patient with Gitelman's syndrome.* Stem Cell Res, 2020. **49**: p. 102110.
